# Decrypting the antisolvent-modulating mechanism in localized high-concentration electrolytes

**DOI:** 10.1093/nsr/nwaf297

**Published:** 2025-07-25

**Authors:** Ruilin Hou, Linlin Zheng, Tianze Shi, Haoyu Li, Shaohua Guo, Haoshen Zhou

**Affiliations:** Center of Energy Storage Materials & Technology, College of Engineering and Applied Sciences, Jiangsu Key Laboratory of Artificial Functional Materials, National Laboratory of Solid State Microstructures, Collaborative Innovation Centre of Advanced Microstructures, Nanjing University, Nanjing 210093, China; Lab of Power and Energy Storage Batteries, Shenzhen Research Institute of Nanjing University, Shenzhen 518000, China; Center of Energy Storage Materials & Technology, College of Engineering and Applied Sciences, Jiangsu Key Laboratory of Artificial Functional Materials, National Laboratory of Solid State Microstructures, Collaborative Innovation Centre of Advanced Microstructures, Nanjing University, Nanjing 210093, China; Lab of Power and Energy Storage Batteries, Shenzhen Research Institute of Nanjing University, Shenzhen 518000, China; Center of Energy Storage Materials & Technology, College of Engineering and Applied Sciences, Jiangsu Key Laboratory of Artificial Functional Materials, National Laboratory of Solid State Microstructures, Collaborative Innovation Centre of Advanced Microstructures, Nanjing University, Nanjing 210093, China; Lab of Power and Energy Storage Batteries, Shenzhen Research Institute of Nanjing University, Shenzhen 518000, China; Center of Energy Storage Materials & Technology, College of Engineering and Applied Sciences, Jiangsu Key Laboratory of Artificial Functional Materials, National Laboratory of Solid State Microstructures, Collaborative Innovation Centre of Advanced Microstructures, Nanjing University, Nanjing 210093, China; Lab of Power and Energy Storage Batteries, Shenzhen Research Institute of Nanjing University, Shenzhen 518000, China; Center of Energy Storage Materials & Technology, College of Engineering and Applied Sciences, Jiangsu Key Laboratory of Artificial Functional Materials, National Laboratory of Solid State Microstructures, Collaborative Innovation Centre of Advanced Microstructures, Nanjing University, Nanjing 210093, China; Lab of Power and Energy Storage Batteries, Shenzhen Research Institute of Nanjing University, Shenzhen 518000, China; Center of Energy Storage Materials & Technology, College of Engineering and Applied Sciences, Jiangsu Key Laboratory of Artificial Functional Materials, National Laboratory of Solid State Microstructures, Collaborative Innovation Centre of Advanced Microstructures, Nanjing University, Nanjing 210093, China

**Keywords:** antisolvent, molecular configuration, ion transport, solid electrolyte interphase, lithium metal battery

## Abstract

Antisolvents are one of the main components in localized high-concentration electrolytes (LHCEs), which are considered merely as diluents to control the macro properties and preserve the anion-dominated solvation structure, but their role in shaping the micromicelle-like structure and interface chemistry remains poorly understood. Here, we utilize LHCEs with trifluorobenzene isomer as antisolvent to investigate the antisolvent polarity-dependent solvation structure, interface chemistry and Li deposition behavior in lithium metal batteries (LMBs). The ‘dragging effect’ of antisolvents on anions can alter the solvation environment, correcting the existing micelle-like solvation structure model of LHCEs. Additionally, the interface adsorption of polar antisolvent negatively impacts the formation of solid electrolyte interphase and ion transport dynamics, thereby influencing the Li deposition behavior. The Li deposition/stripping efficiency in ester-based LHCEs using low-polarity antisolvents is enhanced to 98.55%, as evidenced by the Li||LiFePO_4_ full cell (N/P = 3) achieving 90% capacity retention after 250 cycles. Furthermore, the significant role of high-polarity antisolvent in enhancing the ion conductivity of bulk electrolyte, especially at low temperatures, cannot be ignored. This work provides valuable insights into the intricate role of antisolvents in LHCEs, highlighting their pivotal influence on battery performance and contributing to the advancement of electrolyte design for high-energy-density LMBs.

## Teaser

This work decrypts the influence mechanism of the antisolvent polarity on the solvation structure, interfacial chemistry and lithium metal anode deposition behavior in localized high-concentration electrolyte.

## INTRODUCTION

Localized high-concentration electrolytes (LHCEs) are a cutting-edge solution representing the next generation of advanced liquid electrolytes, designed to support the development of high-energy rechargeable batteries [1–5]. This innovative technology not only facilitates long-term operational stability of highly reactive, energy-dense anodes through the formation of an anion-derived stable inorganic solid electrolyte interphase (SEI), but also addresses the challenge of slow ion transport in high-concentration electrolytes by maintaining low viscosity and a wide working temperature range [6]. Recently, the application of LHCEs has propelled the energy density of lithium metal batteries (LMBs) beyond 500 Wh kg^−1^ [7–9], and they also demonstrate remarkable potential for application under extreme working conditions, including low temperatures [10,11] and high voltages [12,13].

The development and application of diluents are key pathways to enhance the practicality of anionic functional electrolytes. A diluent, typically an antisolvent, does not dissolve lithium salt but is miscible with the main solvent [12,14]. From the perspective of electrostatic potential (ESP) distribution, its ESP_max_ is usually higher than |ESP_min_|, making it a less-than-ideal electron donor [15]. For instance, LHCEs utilize high fluorine ether or ester solvents as antisolvents, which, through the electron-absorption effect of fluorine atoms, evenly disperse the negative charge on the surface of oxygen atoms across both oxygen and fluorine atoms, thereby becoming desirable non-coordinating solvents [16,17]. Consequently, the prevailing consensus on the solvation structure of LHCEs is that the primary solvation sheath of cations is predominantly composed of anions and main solvents, with antisolvents barely involved [18]. The antisolvent, uniformly distributed around the solvated clusters, is merely characterized as ‘dilution’. Additionally, antisolvents with high fluorine content endow the electrolyte with high-voltage resistance, low freezing point and flame-retardant characteristics, which have also been utilized in the design of advanced secondary batteries [10,19–21]. However, for an extended period, researchers have primarily focused on the participation of anions in the primary solvation sheath, as this is crucial for the functionalization of anions in the electrolyte, while the role of antisolvents in regulating microstructure and macro performance has been almost ignored.

**Figure 1.**
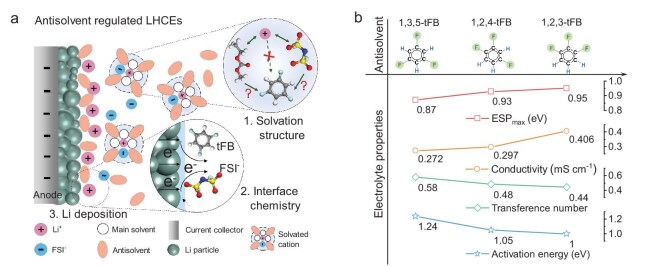
The important role of antisolvents in the properties of LHCEs and energy storage process of LMBs. (a) Schematic illustration of antisolvent regulation of the solvation structure, interface chemistry and Li deposition for LMBs. (b) The electrolyte properties of LHCEs change as the configuration of the antisolvent changes, including ion conductivity, Li^+^ transference number and diffusion activation energy.

In fact, it is imprudent and unscientific to define the role of antisolvents in secondary batteries so simplistically, whether from the perspective of thermodynamics or the systematic of battery design. On the one hand, from a thermodynamic standpoint, antisolvents are not completely non-polar [22]. When mixed with high-concentration electrolytes, a wide range of antisolvent–anion or antisolvent–main solvent interactions must occur within the system, which serve as the theoretical foundation for maintaining the stable existence of short- to medium-range ordered low-entropy clusters in LHCEs. On the other hand, according to the statistics of LHCEs developed thus far, the volume/molar ratio of antisolvent in the electrolyte often exceeds 30% [23]. From a probabilistic perspective, such an abundance of solvent molecules must play a significant role in the core area of energy storage (the electrode–electrolyte interface). Therefore, several unresolved issues remain regarding the role of antisolvents in secondary batteries utilizing LHCEs (Fig. 1a). For example, how does the antisolvent regulate the solvation structure within the region of the second solvation sheath? What role does the antisolvent play in the formation of the interfacial SEI film? And how does the antisolvent existing at the electrode–electrolyte interface impact the dynamics of electrode reaction?

Here, we have developed a series of LHCEs utilizing trifluorobenzene isomers as antisolvents. The distinct molecular configurations of these isomers endow the three antisolvents with varying polarity, which in turn influences the properties of LHCEs. We observed that the antisolvents scarcely interact with the main solvent, yet exhibit a pronounced interaction with anions, a phenomenon that intensifies with increasing antisolvent polarity. This interaction engenders a ‘dragging effect’ on the anions, attenuating the anion–cation interaction within the primary solvation sheath. Therefore, LHCEs formulated with the antisolvent of the lowest polarity facilitate anion decomposition, fostering the formation of LiF-rich SEI films, and culminating in the uniform, dendritic-free Li deposition. Significantly, the electrostatic adsorption of high-polarity antisolvent onto the electrode surface leads to their accumulation at the interface, thereby participating in SEI film formation and impeding ion transport dynamics. Consequently, ester-based LHCEs with low-polarity antisolvent exhibit superior compatibility with Li metal anodes, achieving high average Coulombic efficiency (CE), low overpotential and remarkable cycle stability. The Li||LiFePO_4_ (Li||LFP) full cells with an N/P ratio of 3 maintained 90% of their initial capacity after 250 cycles. This work revised the micellar-like solvation structure model of LHCEs and elucidates the influence mechanism of antisolvent polarity on solvation environment, interface chemistry and Li deposition behavior, offering valuable insights for future research and development in this field.

## RESULTS AND DISCUSSION

### Antisolvent molecular design and regulation of electrolyte properties 

To accurately assess the effects of antisolvents on LHCEs, it is essential to select antisolvents with appropriate molecular structures. In this work, a series of trifluorobenzenes were chosen as antisolvents for the preparation of LHCEs (see Fig. S1 in Supplementary data), including 1,3,5-trifluorobenzene (a-tFB), 1,2,4-trifluorobenzene (b-tFB) and 1,2,3-trifluorobenzene (c-tFB). These selections were made after careful consideration of various potential adverse factors. First, as shown in Table S1, all selected compounds are typical antisolvents that satisfy the requirement of ESP_max_ > |ESP_min_| [15], which indicates that they have weak interactions with Li^+^ ions (see Fig. 1b and Fig. S2) and demonstrate low solubility for lithium salts. Second, the three antisolvents are isomers, which helps eliminate any unknown effects that may arise from differing functional groups. Third, the cyclic structure of these compounds mitigates steric hindrance that could result from variations in molecular configuration of antisolvent, such as those seen in commonly used linear high-fluorinated ether/ester antisolvent (see Fig. S3). Fourth, their chemical properties are stable and do not undergo significant spontaneous chemical reactions with lithium metal. Consequently, the differences among the three trifluorobenzenes, characterized by their distinct molecular configurations, are primarily reflected in their polarity (a-tFB < b-tFB < c-tFB), as shown in Fig. 1b. Subsequently, using ethyl methyl carbonate (EMC) and fluoroethylene carbonate (FEC) as the main solvent, three LHCEs (1 m LiFSI in EMC/tFB/FEC; m/m/m = 3/6/1) were prepared with the aforementioned antisolvents, referred to as a-tFB, b-tFB and c-tFB, respectively (see Fig. S4).

Subsequently, the influence of antisolvent polarity on the physical properties of electrolytes was further investigated. As illustrated in Fig. 1b and Fig. S5, the ionic conductivity of LHCEs increases with the polarity of the antisolvent. Specifically, the conductivity of c-tFB reaches 0.406 mS cm^−1^, compared to 0.272 mS cm^−1^ for a-tFB and 0.297 mS cm^−1^ for b-tFB. Furthermore, the ion-diffusion activation energy for the three electrolytes (see Fig. 1b and Fig. S6) indicates that high-polarity antisolvent enhances ion transport within the electrolyte, resulting in a lower ion-diffusion activation energy (*Ea*_c-tFB_ = 1.00 eV). However, a-tFB exhibits the highest Li^+^ transference number of 0.58, which is greater than that of b-tFB (0.48) and c-tFB (0.44), as demonstrated in Fig. 1b and Figs S5a and S7. Moreover, the theoretical calculation results shown in Fig. S8 also exhibit the same trend of change. The contrasting trends of ionic conductivity and Li^+^ transference number with increasing antisolvent polarity suggest that the enhancement of ionic conductivity is not merely due to an increase in the degree of dissociation of anions and cations (i.e. the number of free ions), but rather a complex interplay of changes in the solvation structure and properties of the electrolyte. Additionally, the polarity of the three electrolytes has a relatively minor impact on the voltage window, with the oxidation decomposition potentials of all electrolytes exceeding 5.0 V vs. Li/Li^+^ based on Al foil electrode or 4.5 V vs. Li/Li^+^ based on carbon electrode (see Fig. S10). In summary, the alterations in macroscopic properties clearly demonstrate that the antisolvents regulate the microscopic solvation structure of electrolytes, which warrants further in-depth research.

### Rationales for antisolvent-controlled solvation structure

Previous research has demonstrated that LHCEs have a unique micelle-like solvation structure [18], in which the main solvent functions as a surfactant between the antisolvent and the charge-carrying ions. This model completely ignores the possible role of polar antisolvents, and fails to clarify whether antisolvents interact with anions or solvents in the primary solvation sheath. In theory, the influence trends of antisolvent–anion interaction and antisolvent–main solvent interaction on the primary solvation structure are opposite, which warrants further in-depth investigation. Therefore, the various interactions that may exist within the micelle-like solvation structure of LHCEs, including cation–anion interactions, cation–main solvent interactions, anion–antisolvent interactions and main solvent–antisolvent interactions, have been extensively studied using Fourier transform infrared spectroscopy (FT-IR), Raman spectroscopy and molecular dynamics simulations.

**Figure 2.**
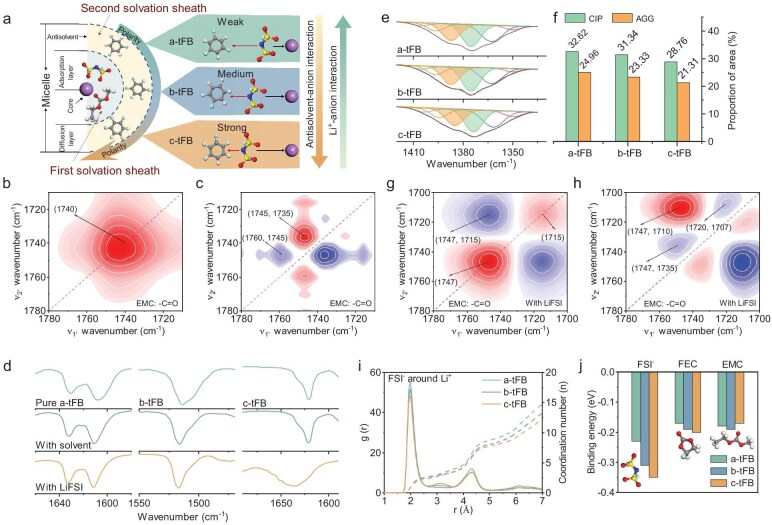
The regulatory effect of antisolvents on solvation structure of LHCEs. (a) Schematic illustration of the micelle-type solvation structure of LHCEs and the regulatory effect of antisolvent on it. (b) Synchronous and (c) asynchronous 2D IR correlation spectra for the EMC mixed with different antisolvents in the 1780–1710 cm^−1^ region. (d) FT-IR spectra of different pure antisolvents, antisolvents mixed with main solvents and LHCEs. (e) FT-IR spectra of FSI^−^ in LHCEs with different antisolvents. (f) The area proportion of CIP and AGG characteristic peaks in the entire peak ranging from 1420 to 1335 cm^−1^. (g) Synchronous and (h) asynchronous 2D IR correlation spectra for the EMC in LHCEs with different antisolvents in the 1780–1700 cm^−1^ region. (i) Radial distribution function g(r) analyses for the Li^+^–FSI^−^. (j) The binding energies between different antisolvents and FSI^−^, FEC and EMC.

First, the dynamic behaviors of dipole interactions between main solvent and antisolvent were elucidated using 2D infrared correlation spectroscopy (2D IR COS), with the polarity of the antisolvent serving as an external perturbation, which includes synchronous 2D COS and asynchronous 2D COS. The former reflects the correlation trends of different characteristic peaks under external perturbations, while the latter analyzes the correlation between each sub-peak after decoupling [24,25]. In 2D IR COS, positive and negative cross peaks are represented by red and blue colors, respectively. As shown in Fig. 2b, a single auto peak at approximately 1740 cm^−1^ is observed in the synchronous 2D COS of EMC (–C=O) within the range of 1710–1780 cm^−1^, confirming that EMC is not coordinated with the antisolvents. Within the same range, the asynchronous 2D COS of EMC (Fig. 2c) reveals that the two shoulder peaks (1735 and 1760 cm^−1^) weaken relative to the main peak (1745 cm^−1^), indicating that this characteristic peak gradually sharpens with increasing antisolvent polarity. This phenomenon is attributed to the entropy increase caused by the introduction of antisolvents, which diminishes the interaction between EMC molecules. Additionally, the 2D IR COS results of FEC (–C=O) indicate that FEC is less affected by the polarity of the antisolvent, likely due to its low content in hybrid solvents (see Fig. S11). Furthermore, Raman spectroscopy demonstrated that the characteristic peaks of EMC and FEC remained unchanged after the introduction of the antisolvent, suggesting that the interaction between the main solvent and the antisolvent can be considered negligible (see Fig. S12a–c). As illustrated in Fig. 2d and Fig. S13, although the main solvent and antisolvent are miscible, the peak A (in-plane cyclic tensile vibration characteristic peaks, 1500–1650 cm^−1^) and peak B (aromatic hydrogen out-of-plane bending-ring skeleton vibration characteristic peak, ∼850 cm^−1^) of the three antisolvents [26] only exhibited slight shifts, indicating weak dipole interactions between them. Overall, the analysis presented above demonstrates that the dipole interaction between the main solvent and antisolvent is weak.

After further dissolving the LiFSI in the mixed solvent, the FT-IR spectra have undergone significant changes involving main solvent, antisolvent and Li salt. First, regarding a-tFB and b-tFB, there is no significant change in peak A of the antisolvent (Fig. 2d), while peak B has widened and shifted towards lower wavenumbers (see Fig. S13), indicating weak hydrogen-bonding interactions formed between the ions and the antisolvent [27]. When the antisolvent is c-tFB, which has maximum polarity, both peak A and peak B are widened and shifted towards higher wavenumbers (Fig. 2d and Fig. S13), which is attributed to the conjugated interactions between the anions and c-tFB [28].

The FT-IR spectral deconvolution results for FSI^−^ indicate that the anions exist in the form of coordinated FSI^−^ (1390 cm^−1^) in the three electrolytes (Fig. 2e and Fig. S14), confirming that the anions participate in the primary solvation structure of Li^+^, which is a typical solvation structure of LHCEs [29,30]. The peak area ratio of corrugated ion pairs (CIPs) and aggregated ions (AGG) (775 cm^−1^) gradually decreases as the polarity of the antisolvent increases (Fig. 2f). Furthermore, the deconvolution of the Raman characteristic peaks of FSI^−^ indicates that as the polarity of the antisolvent increases, the proportion of CIPs and AGG decreases (see Figs S12d–f and S15a), which is consistent with the FT-IR results. Additionally, the synchronous 2D COS of EMC (–C=O) at 1700–1780 cm^−1^, splits into free EMC (1715 cm^−1^) and coordinated EMC (1747 cm^−1^) after introduction of LiFSI, showing a negative correlation between them (Fig. 2g). The asynchronous 2D COS reveals that the characteristic peak of coordinated EMC is enhanced, indicating that the Li^+^–EMC interaction increases with the increasing polarity of the antisolvent (Fig. 2h). This phenomenon occurs because the weak interaction between anions and cations in the c-tFB electrolyte diminishes the charge-shielding effect of anions on cations, thereby intensifying the interaction between Li^+^ and the main solvent. Furthermore, the Li^+^ radial distribution functions (RDFs) of the three electrolytes demonstrate that the Li^+^ primary solvation sheaths are mainly composed of anions and a small amount of the main solvent, with no presence of the antisolvent (see Fig. S16). Moreover, as the polarity of the antisolvent increases, the anionic coordination number decreases while the amount of the main solvent increases, which aligns with the FT-IR results (Fig. 2i). As shown in Fig. 2j, the binding energies between the main solvent or anions and the three antisolvents were further calculated. The results indicated that, on the one hand, the anion–antisolvent interaction is stronger than the main solvent–antisolvent interaction. On the other hand, as the polarity of the antisolvent increases, the anion–antisolvent interaction strengthens. The chemical shift of ^1^H in a/b/c-tFB shifts up-field in the presence of LiFSI (see Fig. S17), suggesting the shielding effect originated from the antisolvent–anion interaction. Moreover, as the polarity of the antisolvent increases, the chemical shift is more obvious, meaning a stronger antisolvent–anion interaction. Furthermore, the increase in the distance between cations and anions and the primary solvation size (see Fig. S16d) further demonstrates the ‘dragging effect’ of the strongly polar antisolvent on the solvation structure. It should be emphasized that, compared with the strong interaction between cations and anions, the weaker antisolvent–anion interaction can only have a relatively mild regulatory effect on the solvation structure (see Fig. S15b).

Thus, the solvation structure of the LHCEs and the regulatory mechanism of the antisolvent have been clarified. Specifically, as illustrated in Fig. S18, typical LHCEs utilizing trifluorobenzene as the antisolvent exhibit Li^+^–FSI^−^ interaction, Li^+^–main solvent interaction and anion–antisolvent interaction. Among these, the anion–antisolvent interaction plays a crucial role in regulating the solvation structure (Fig. 2a). Specifically, this interaction will make the antisolvent produce a ‘dragging effect’ on the anions in the primary solvation sheath. As expected, despite the ‘dragging effect’ occurring outside the primary solvation cluster, it still weakens the Li^+^–anion interaction and promotes the Li^+^–main solvent interaction, which may lead to a poor SEI film derivation effect and high desolvation energy. Therefore, for fluorinated aromatic antisolvent, low-polarity antisolvent is more ideal for LHCE design, which can make LHCEs more localized.

### Antisolvent-dependent solid electrolyte interphase

**Figure 3.**
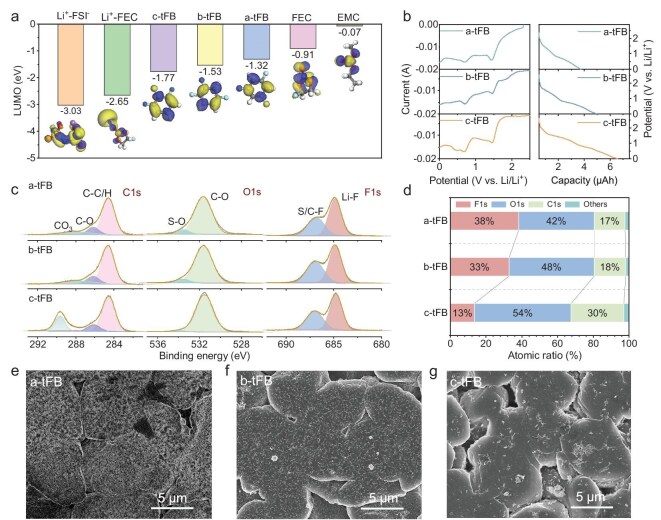
Antisolvent-dependent SEI film derivation mechanism and Li deposition morphology. (a) LUMO energy levels of various species in LHCEs with different antisolvents. (b) The first discharge CV and GCD profiles of Li||Cu cells using LHCEs with different antisolvents. (c) C1s, O1s and F1s spectra of Cu current collectors after 10 cycles of Li deposition/stripping in different LHCEs. (d) Atomic ratio in SEI film formed in different LHCEs. (e–g) SEM images of Li anode deposited in a-tFB (e), b-tFB (f) and c-tFB (g), respectively.

In addition to ion transport properties, the derivatization ability of SEI films is also a crucial indicator of electrolyte performance. The sequence and quantity of species decomposition within the electrolyte determine the component and structure of the SEI film. First, the lowest unoccupied molecular orbital (LUMO) of various species in the electrolyte was examined using density functional theory (DFT) to explore their thermodynamic reduction potentials. As illustrated in Fig. 3a, the reduction potential decreases in the following order: coordinated FSI^−^ ˃ coordinated FEC ˃ c-tFB ˃ b-tFB ˃ a-tFB ˃ free FEC ˃ free EMC. This indicates that antisolvents are more prone to decomposition than main solvents. In the absence of a dense SEI film formation, antisolvents will also decompose. Moreover, the decomposition processes of electrolytes can be observed in the initial cyclic voltammogram (CV) discharge curves and the galvanostatic charge–discharge (GCD) curves (Fig. 3b) of Li||Cu cells. Evidently, as the potential decreases, the three electrolytes undergo two significant decompositions: the reduction of coordinated FSI^−^ at approximately 1.5 V vs. Li/Li^+^ [31] and the decomposition of coordinated FEC [32] and other organic solvents below 1.0 V vs. Li/Li^+^. Furthermore, as the polarity of the antisolvent increases, the ratio of anion decomposition to organic solvent decomposition decreases, as evidenced by the peak current ratio of CV curves and the capacity ratio in the GCD curves (see Fig. S19). Notably, the LHCEs with c-tFB as the antisolvent exhibit a distinct reduction peak at 0.5 V vs. Li/Li^+^, which corresponds to the reduction of c-tFB (Fig. 3b) [33]. Based on the above analysis, it can be concluded that LHCEs with high-polarity antisolvents tend to form SEI films primarily through the decomposition of organic solvents (both the main solvent and antisolvent). Further, through adjusting the electrolyte composition and testing their CE, it can be seen that FEC in the electrolyte and the antisolvent cooperatively optimize the SEI film (see Fig. S36). Specifically, FEC plays a role in stabilizing the interface between the ester solvent and lithium metal in the early stage of the SEI film formation. However, the long-term stability of its structure still relies on the solvation structure regulated by the antisolvent to generate more inorganic LiF.

Combined with the solvation structure analysis above, the formation process of SEI film was further investigated using *in situ* electrochemical impedance spectroscopy (EIS) conducted on Li||Cu cells at various charging and discharging stages. The typical formation of the SEI film in Li||Cu cells can be divided into three stages: the electrolyte decomposition process [Stage 1, open circuit voltage (OCV) ∼0 V]; the initial Li deposition process (Stage 2, <0 V) and stable Li deposition/stripping process (Stage 3) [34]. In Stage 1, the EIS plot displays only a single semicircle, which corresponds to the charge transfer associated with the electrolyte decomposition process (see Figs S20a and S21a). This is a Faradaic reaction characterized by slow kinetics, exhibiting a longer relaxation time of 100 s (see Fig. S22a). As shown in Fig. S20a and b, compared to the voltage profile in high-polarity c-tFB electrolyte, the discharge time is shorter and the charge transfer impedance increases more rapidly in the a-tFB electrolyte, which indicates a lower amount of decomposition and a higher decomposition potential in the a-tFB electrolyte. The observed behavior is attributed to the stronger interactions between anions and cations in the a-tFB electrolyte, as demonstrated previously. When the potential is further reduced below 0 V (see Fig. S20c), Li metal begins to nucleate and grow on the Cu substrate and continues to form SEI film during Stage 2 (see Figs S21b and S22d and e). At this stage, a dense and complete SEI film has not yet fully formed. Consequently, the ionic conductivity of the electrolyte becomes the primary rate-limiting factor, resulting in higher overpotential (impedance) for the Li||Cu cell in the a-tFB electrolyte (see Figs S20d and S21b). In the subsequent process, during the initial seven cycles of charging and discharging, a dense SEI film gradually forms. Although the a-tFB electrolyte exhibits lower ionic conductivity compared to the c-tFB system, its reduced SEI film resistance (*R*_SEI_) and accelerated interfacial desolvation kinetics (*R*_des_) collectively enable enhanced ion transport efficiency (see Fig. S22f and g). As a result, the overpotential for Li deposition (ion transport impedance) in the a-tFB electrolyte decreases compared to that in the c-tFB electrolyte (see Fig. S20e and f). This improvement is attributed to the SEI film derived from a-tFB, which contains a higher proportion of inorganic components, thereby facilitating more efficient ion transport.

Furthermore, the components of the SEI films derived from the three electrolytes were analyzed using X-ray photoelectron spectroscopy (XPS). As shown in Fig. 3c, all SEI films formed in the three electrolytes exhibit an obvious Li–F peak (684.5 eV) in the F1s spectra and a C–O_3_ peak (289.5 eV) in the C1s spectra, indicating that the SEI films contain two inorganic substances: LiF and carbonate. Notably, the relative peak ratios of C/S–F to Li–F and C–O_3_ to C–C/H increase with the polarity of antisolvents. Additionally, the relative atomic contents of F, C and O in the SEI films were calculated, as shown in Fig. 3d. The F content decreases from 38% in a-tFB to 13% in c-tFB. Moreover, the cycled Li surface maintains SEI compositional characteristics consistent with above findings (see Fig. S23). All evidence suggests that LHCEs with low-polarity antisolvents lead to the formation of LiF-rich SEI films, which are considered ideal for Li metal anodes [35]. Although the antisolvent also contains F atoms, it is evident that the C content is higher, and the stable benzene ring may provide more organic compounds for the formation of the SEI film. Furthermore, cryogenic transmission electron microscopy (cryo-TEM) images show that with the enhancement of the polarity of the antisolvent, the SEI film on the deposited Li metal surface becomes thicker (see Fig. S24a–c). Specifically, the average thickness of the SEI film generated in a-tFB is 36.65 nm, which is thinner than that in b-tFB (41.30 nm) and c-tFB (51.35 nm). There are also a large number of inorganic crystal particles in the SEI film derived from a-tFB (see Fig. S24d). In sharp contrast, the SEI film derived from c-tFB is almost entirely composed of amorphous organic substances (see Fig. S24f). Figure S25 presents the quantification results of SEI dissolution using a method previously established before by the Reza Younesi group [36]. The results indicate that the average capacity loss after different time pauses increases as the polarity of antisolvent rises, demonstrating that the dissolution of the SEI film weakens as decreasing the polarity of the antisolvents. This phenomenon occurs because of the low polarity of the a-tFB electrolyte and its derived SEI being rich in inorganic substances.

The LiF-rich SEI film is advantageous for facilitating ion transport and inhibiting dendritic growth, theoretically. To this end, the morphologies of the deposited Li were examined using scanning electron microscopy (SEM) (Fig. 3e–g). As shown in Fig. 3e and Fig. S26a, mossy Li deposits form in the a-tFB electrolyte, exhibiting a smooth surface with fewer cracks. As the polarity of the antisolvent increases, the deposited Li becomes looser and displays edges and cracks (Fig. 3f and g, Fig. S26b and c). Clearly, LHCEs with lower-polarity antisolvents demonstrate improved compatibility with Li metal anodes.

### Antisolvent-modulated lithium deposition interface dynamics

**Figure 4.**
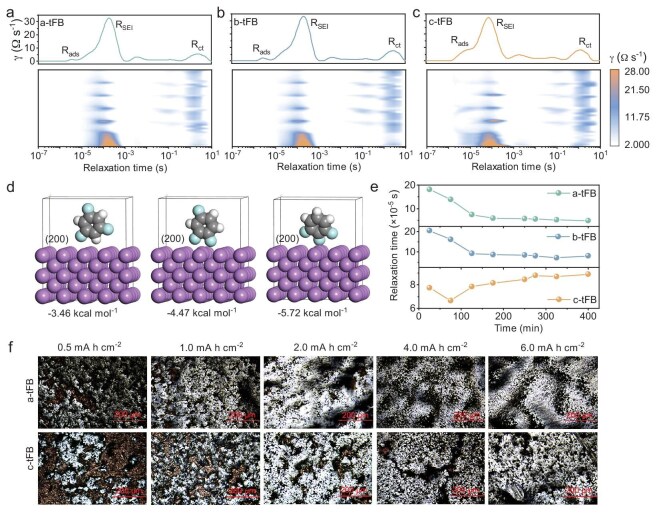
The interface regulation mechanism of antisolvent on Li deposition behavior. *In situ* DRT mapping of the first three cycles of Li||Li cells using a-tFB (a), b-tFB (b) and c-tFB (c), respectively. (d) The adsorption energy of different antisolvent molecules on the Li metal (002) crystal plane. (e) The relaxation time corresponding to the SEI film formation process in different electrolytes with increasing deposition time. (f) Optical photographs of lithium deposition morphology on Cu foil surface under different deposition capacities in different electrolytes (image size: 650 μm × 425 μm).

Indeed, the interface environment at the lithium anode directly influences the ion transport kinetics, interface chemistry and the processes of lithium deposition/stripping. As one of the main components in the electrolyte, the characteristics of the antisolvent play a pivotal role in modulating the interface environment. To explore this, Li||Li symmetric cells were monitored using *in situ* distribution of relaxation times (DRT) during the initial three cycles, which can classify different electrochemical processes by identifying their local maxima in a continuous distribution function [37]. Typically, the ion transport during Li deposition/stripping processes primarily include the interface adsorption impedance (R_ads_, ∼10^−5^ s), the SEI impedance (R_SEI_, ∼10^−4^ s) and charge transfer impedance (R_ct_, 1–10 s), as depicted in Fig. 4a–c [38,39]. During the initial stage of Li deposition, as the polarity of the antisolvent rises, the role of R_ads_ becomes more pronounced due to the ion transport being impeded by the lithiophobic antisolvent adsorbed on the surface. Further theoretical calculations demonstrate that, in comparison to a-tFB or b-tFB with low polarity, c-tFB is more readily adsorbed onto the surface of Li metal (Fig. 4d and Fig. S27). This phenomenon can be attributed to the more robust Coulomb force existing between the high-ESP_max_ antisolvent and the anode. Strong interface adsorption leads to greater interface enrichment, which influences the formation of interface SEI film, and subsequently modulates the Li deposition behavior. As shown in Fig. 4a and b, the R_SEI_ of the Li||Li cell employing a-tFB and b-tFB electrolytes exhibits a steady decline over time as the deposition process continues. In contrast, while the impedance of Li||Li cells in c-tFB also reduces with increasing deposition time, its relaxation time oscillates within a specific range, which may be attributable to the recurring rupture and reformation of the SEI film (Fig. 4c). In addition, as the discharge time increases, the relaxation time of R_SEI_ in c-tFB rises, whereas those in a-tFB and b-tFB gradually decline and approach stability (Fig. 4e and Fig. S28). This phenomenon can be attributed to the fact that the surface adsorption and lower LUMO energy level of c-tFB facilitate its decomposition into SEI films, resulting in low ionic conductivity. The observed initial decline in relaxation time primarily originates from the electrochemical self-cleaning of the passivation layer on Li foil surfaces during early discharge stages. After the complete formation of the SEI film, the R_SEI_ in c-tFB is the largest among all three electrolytes (see Fig. S29). Moreover, the desolvation impedance at a relaxation time of 10^−1^ s only emerges during the exchange of positive and negative current, a phenomenon attributable to changes in the external electric field. With increasing antisolvent polarity, the desolvation-related resistance of Li^+^ significantly increases (see Fig. S29), as evidenced by DRT analysis in Li||Cu cells (see Fig. S22). Both experimental and computational results directly confirm this conclusion (see Figs S30 and S31). Evidently, the antisolvents modulate the Li deposition behavior not only by affecting the solvation structure of the electrolyte and the SEI structure/component but also their unique interface adsorption properties.

The morphology of Li deposition in a-tFB and c-tFB with different deposition capacity was then studied by optical microscopy. As shown in Fig. 4f, when the deposition capacity is 0.5 mAh cm^−2^, there is more Li nucleation in a-tFB and they are more uniform than those in c-tFB. Combined with the Li deposition voltage profiles after forming a stable SEI film (see Fig. S32), it can be seen that the Li nucleation overpotential in c-tFB (63.6 mV) is larger than that in a-tFB (48.4 mV). In other words, the highly polar antisolvent system is not conducive to the uniform nucleation of Li, which may be related to the SEI film with low ionic conductivity and at the lithiophobic antisolvent enrichment interface. At a deposition capacity of 2.0 mAh cm^−2^, Li almost completely covers the Cu substrate in a-tFB, while there are still some undeposited areas on the Cu foil in c-tFB. As the deposition capacity increases to 4 mAh cm^−2^, the uneven island-like Li metal in c-tFB gradually connects, but obvious cracks remain. In contrast, the deposited lithium on Cu substrate in a-tFB has grown more compact. When the capacity reaches 6 mAh cm^−2^, the deposited Li on Cu substrate in c-tFB also becomes denser, but it is still rougher than that in a-tFB. Even at a larger observation area (see Fig. S33), the results are consistent with the above description. In addition, the adsorption capacity at the electrode interface was calculated by CV after the Li||Li cell was cycled for 10 cycles. It can be observed that the adsorption capacity increases with the increase of antisolvent polarity, suggesting that the surface of Li electrode becomes rougher and the specific surface area larger (see Fig. S34).

### Performance of Li metal anodes and Li||LFP full cells

**Figure 5.**
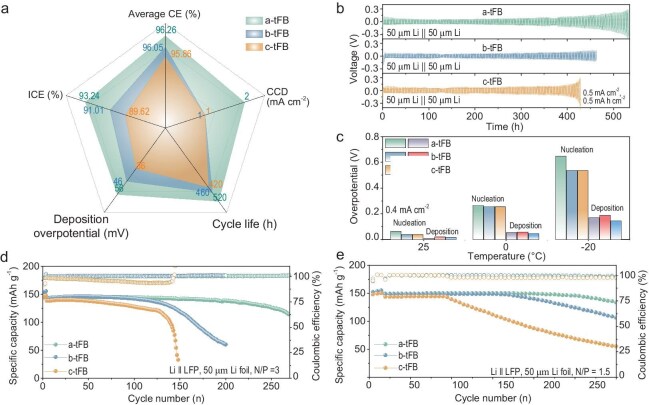
Electrochemical performance of Li metal anode and Li||LFP full cells at different LHCEs. (a) Radar plots of the electrochemical performance for Li metal anode in different LHCEs, involving ICE, average CE, deposition overpotential, CCD and cycle life. (b) Cycle performance of Li||Li cells with limited Li (50 μm) in different LHCEs. (c) Comparison of Li nucleation and deposition potentials on Cu foil in different electrolytes at different temperatures. The cycling performance of (d) Li||LFP full cells (N/P = 3) and (e) Li||LFP full cells (N/P = 1.5) using LHCEs with different antisolvents.

To explore the compatibility of a Li metal anode with LHCEs containing different antisolvents, Li||Cu cells were assembled using these electrolytes for testing. The Li deposition/stripping CEs were determined using an improved Aurbach method [40], as shown in Fig. S35a. The CE for Li deposition in the three electrolytes exceeds 98.50%, with a slight decrease observed as the polarity of the antisolvent increases, while it is significantly higher than that without trifluorobenzene (see Fig. S36a). Moreover, during long cycling tests of Li||Cu cells, compared with the average CEs of both b-tFB (97.22% at 0.2 mA cm^−2^, 96.05% at 0.5 mA cm^−2^) and c-tFB (97.04% at 0.2 mA cm^−2^, 95.86% at 0.5 mA cm^−2^), the a-tFB electrolyte achieved smaller fluctuations and a higher average CE (97.45% at 0.2 mA cm^−2^, 96.26% at 0.5 mA cm^−2^) for Li deposition/stripping over 200/250 cycles (see Figs S35b and S37). Additionally, as shown in the potential profiles of symmetric cells using thin Li foils (50 μm) at 0.5 mA cm^−2^ and 0.5 mA h cm^−2^ (Fig. 5b), all the cells in a-tFB, b-tFB and c-tFB display small overpotentials (∼25 mV) for the first 400 h of cycling. After 420 h, the cell in c-tFB fails first. In contrast, the cell in a-tFB does not fail until 540 h later. Similarly, with different current densities of 0.2 and 1.0 mA cm^−2^, the cycle life of the Li||Li cells using a-tFB is longer than that using the other two antisolvents (see Fig. S38). The a-tFB electrolyte system exhibits enhanced SEI film stability (R_SEI_ increase < 2 Ω after 200 cycles) and optimal dendrite suppression efficacy, as evidenced by stable overpotential profiles (<5 mV fluctuation) throughout 200 cycles (see Fig. S39). Meanwhile, the cell in a-tFB achieves a larger critical current density (CCD) of 2.0 mA cm^−2^ compared to those in b-tFB and c-tFB (see Fig. S35c). In summary, LHCEs with low-polarity antisolvents demonstrate improved compatibility with Li metal anodes, characterized by higher initial cycle CE, reduced deposition overpotential, increased average CE, larger CCD and extended cycle life (Fig. 4a). It should be emphasized that the a-tFB-based LHCEs we developed have achieved the best compatibility with lithium metal anodes within the category of ester-based electrolytes (Table S2).

Although the electrochemical performance of Li||Cu or full cells at room temperature demonstrates the excellent compatibility of Li metal anodes with LHCEs, it does not fully reflect the regulatory effects of antisolvents. To address this, the electrochemical performance of Li metal anodes in LHCEs was further evaluated at low temperatures (0°C). As shown in Fig. S40, the cell using a-tFB exhibits a smaller Li deposition/stripping overpotential compared to the cell using c-tFB. Additionally, the voltage profiles of cells with c-tFB show more pronounced fluctuations, which become more severe as the current density increases. This is attributed to the higher ionic conductivity of inorganic-rich SEI formed by a-tFB electrolyte. Meanwhile, when the temperature drops to 0°C, the magnitude of CE loss intensifies with the increasing polarity of the antisolvent (see Fig. S41). However, as illustrated in Fig. 5c, as the temperature decreases, both the lithium nucleation overpotential and the deposition potential during the first discharge cycle increase significantly (Fig. 5c). Clearly, at low temperatures, c-tFB, which has higher conductivity, facilitates lower nucleation and deposition overpotential for the first cycle. In LHCEs using b-tFB as antisolvent with relatively high R_SEI_ and low ionic conductivity at −20°C, the deposition overpotential is the highest. Therefore, when considering the low operating temperatures, it is essential to take account of the opposing influence of antisolvent polarity on conductivity and SEI impedance.

To further demonstrate the excellent Li metal anode compatibility of LHCEs, the Li metal full cell was assembled with a high-loaded LFP cathode (∼20 mg cm^2^, N/P ratio is 3) and a-tFB electrolytes, which achieved excellent cycling stability of 90% initial capacity retention after 250 cycles at 0.5°C (Fig. 5d). Simultaneously, the CE of the full cell is close to 100% in the whole cycle life. In contrast, the capacity of the full cell with b-tFB electrolyte began to decline rapidly after 125 cycles, while the capacity retention rate of the full cell using c-tFB is worse under the same conditions. Their respective GCD curves (see Fig. S42) showed more clearly that the polarization of the full cell gradually increased with the increase of the number of cycles. With the increase of antisolvent polarity, this phenomenon occurs earlier, which is caused by the rapid depletion of limited Li metal anode. The initial CE (ICE) of the full cell (see Fig. S43) with a-tFB electrolyte is 95.11% at 0.2°C, which is higher than that using b-tFB (93.11%) and c-tFB (83.7%). Moreover, when the N/P ratio of the full cells was reduced to 1.5 (Fig. 5e), the Li||LFP cells with a-tFB electrolyte could still maintain 90% of the initial capacity after 60 cycles, while the cells with c-tFB began to decay rapidly after 18 cycles, which is caused by the high discharge depth of the Li anode at low N/P ratio. In fact, this can also be demonstrated by the ultra-long cycle stability of the Li||LFP full cells without limiting N/P ratio (see Fig. S44). Obviously, the effect of antisolvent polarity on Li metal anodes is further amplified under the harsh conditions of the full cell. In fact, under the condition of a limited Li anode, the full cell using a-tFB-based LHCEs has achieved extremely excellent cycling stability compared with that with other ester-based electrolytes summarized in Table S3.

### Overall evaluation of antisolvent and the structure–property correlations

**Figure 6.**
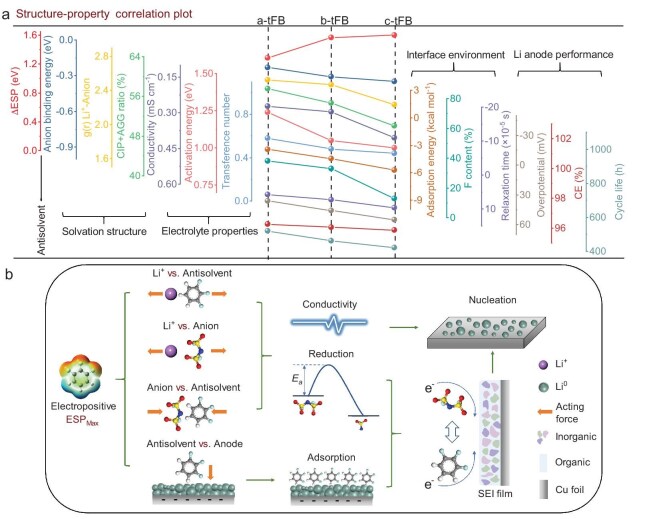
Summary and overall evaluation of antisolvent. (a) Structure–property relationship plot of antisolvent, Li^+^ solvation environment, electrolyte properties, interface chemical and Li metal anode performance. The axes are shown in gradient height and each axis corresponds to the line chart with the same color. (b) Schematic diagram of the regulatory mechanism of antisolvent on the solvation structure, interface chemistry and Li deposition behavior of LMBs.

To date, we have conducted a comprehensive evaluation of the regulatory role of antisolvents in LHCEs from three perspectives: solvation environment; electrolyte properties; and interface chemistry, as well as Li deposition behavior. As plotted in Fig. 6a, these four pivotal aspects, which are crucial for the performance of Li metal batteries, particularly the Li metal anode, have been cross-validated through 13 parameters/properties. These parameters exhibit a certain regularity with the polarity of the antisolvent, offering a structure–property correlation between the electrolyte and battery performance. The underlying logic and relationships are as follows.

Firstly, the change in polarity of the antisolvents directly impacts their binding energy with anions. Consequently, the strengthened anion–antisolvent interaction encourages more anions to dissociate from Li^+^, as evidenced by the decreased probability of anion distribution around Li^+^ and the ratio of AGG and CIP. This clearly demonstrates that, despite the absence of direct interaction between antisolvents and Li^+^, they can indirectly modulate the solvation environment by interacting with anions (Fig. 6b). Secondly, high-polarity antisolvents enhance the dissociation of lithium salts by engaging with anions, resulting in increased ion conductivity and reduced diffusion activation energy of the LHCEs. The interaction between anions and antisolvents facilitates anion transport, thereby reducing the transference number of Li^+^.

Third, antisolvents significantly influence ion transport and Li deposition through interface adsorption and the formation of SEI film. On the one side, high-polarity antisolvents exhibit strong adsorption energy on the Cu substrate surface. The accumulation of antisolvent at the interface hinders ion transport during the initial deposition process and facilitates the formation of an organic SEI film (Fig. 6b). On the other side, low-polarity antisolvents help maintain the anion-dominated primary solvation structure, leading to a LiF-rich SEI film with a shorter relaxation time. Moreover, an interphase with high ionic conductivity accelerates the desolvation process. Ultimately, by tuning the solvation structure of LHCEs and the SEI film at the interface, antisolvents significantly regulate the performance of Li metal batteries, particularly affecting the Li metal anode. Specifically, Li metal anodes achieve lower deposition overpotential, higher deposition/stripping CE and improved cycle stability in the LHCEs with low-polarity antisolvents. Thus, a clear and logical pathway for regulating the electrolyte for Li metal batteries through antisolvents has been fully established (Fig. 6b).

Obviously, the regulation of the properties of LHCEs and the performance of LMBs by antisolvents is influenced by the antisolvent–anion interaction, as well as the adsorption of antisolvents at the anode interface, both of which depend on molecular polarity. In LHCEs, the competitive coordination of cations and antisolvents with anions not only regulates the thermodynamic potential for the decomposition of anions, but also affects the ion transport characteristics within the electrolyte. The electrostatic adsorption of polar antisolvents on the electrode surface alters the electric double-layer environment, subsequently impacting the derivation reactions of SEI film at the interface and the ion transport during the initial stages, thereby directly influencing the lithium deposition behavior. Consequently, it is crucial for the design of advanced LHCEs to carefully engineer the structure of antisolvents to modulate their polarity in accordance with the operating conditions of LMBs. It is important to note that, in theory, interactions among all types of polar molecules (ions) can facilitate structural regulation in the peripheral solvation layer and play a significant role at the interface. The anion–antisolvent interaction discussed in this work specifically pertains to fluorinated aromatic hydrocarbon antisolvents, which may not be applicable to antisolvents with different molecular configurations; however, the underlying research concept is broadly applicable. Therefore, greater attention should be given to the design of antisolvents.

## CONCLUSION

In conclusion, we have successfully elucidated the influence mechanism of antisolvent polarity in LHCEs on the solvation structure, interface chemistry and Li deposition behavior in LMBs. It is demonstrated that the antisolvent surrounding the primary solvation clusters can modulate the solvation structure through interactions with anions. A low-polarity antisolvent is more conducive to maintaining strong cation–anion interaction and forming an anion-derived, LiF-rich SEI film. In addition, the preferential adsorption of high-polarity antisolvent on the Li metal surface promotes the decomposition of antisolvent, forming an organic SEI film with poor conductivity and impeding the interface ion transport, thus ultimately affecting the Li deposition behavior. Notably, the positive effect of high-polarity antisolvent on the conductivity of bulk electrolytes has a beneficial impact on battery performance in low-temperature application scenarios. Therefore, the key to customizing the properties of LHCEs lies in reasonably adjusting the polarity of the antisolvent to balance the electrolyte conductivity and the interfacial resistance of SEI film. Due to the minor disturbance of the anion-dominated solvation structure by the a-tFB diluent, an inorganic-rich SEI film is generated, enabling the Li||LFP full cell with limited Li (50 μm) and a high-load cathode to achieve an extended cycle life. This study enhances the understanding of the antisolvent effect in LHCEs, revises the solvation structure model of LHCEs, and highlights the crucial influence of polar-molecular interface adsorption on interface chemistry and Li deposition behavior.

## Supplementary Material

nwaf297_Supplemental_File
