# Supplementary material for: Decrypting the antisolvent-modulating mechanism in localized high-concentration electrolytes
Source: Natl Sci Rev. 2025 Jul 25;12(9):nwaf297. doi: 10.1093/nsr/nwaf297 (PMC12449081; doi:10.1093/nsr/nwaf297)
Supplement: nwaf297_Supplemental_File [file nwaf297_supplemental_file.pdf]

Supporting Information

Decrypting the antisolvent-modulating mechanism in localized high-concentration electrolytes

Ruilin Hou[1,2], Linlin Zheng[1,2], Tianze Shi[1,2], Haoyu Li[1,2], Shaohua Guo[1,2] * and Haoshen Zhou,[1] *

[1] Center of Energy Storage Materials & Technology, College of Engineering and Applied Sciences, Jiangsu Key Laboratory of Artificial Functional Materials, National Laboratory of Solid State Microstructures, Collaborative Innovation Centre of Advanced Microstructures, Nanjing University, Nanjing 210093, China

[2] Lab of Power and Energy Storage Batteries, Shenzhen Research Institute of Nanjing University, Shenzhen 518000, China

* Corresponding author: E-mail: hszhou@nju.edu.cn; shguo@nju.edu.cn

Experimental section

Electrolyte preparations:

The antisolvents of 1,3,5-trifluorobenzene (a-tFB, 99.0%), 1,2,4-trifluorobenzene (b-tFB, 99.0%) and 1,2,3-trifluorobenzene (c-tFB, 99.0%) were purchased from Aladdin. LiFSI (99.8%), fluoroethylene carbonate (FEC, 99.0%) and ethyl methyl carbonate (EMC, 99.0%) were purchased from Dodo Chem. Typical 1 m (mol kg^{-1}) LHCEs are obtained by dissolving 0.187 g LiFSI in a mixed solvent of FEC/EMC/antisolvent (0.1/0.3/0.6 mg) with mass ratio of 1/3/6. All the LHCEs were made and stored in an argon-filled glovebox (H_2O<0.01 ppm, O_2<0.01 ppm).

Electrode preparation and cell fabrication:

The Cu foil electrodes were washed three times sequentially with dilute hydrochloric acid, deionized water, and acetone. The LFP cathode is made by coating lithium iron phosphate slurry (LFP/PVDF/Super P=8/1/1, m/m/m) on an Al foil current collector, which is dried in a vacuum oven at 120 ℃. The loading of LFP is ~20 mg cm^{-2}. The Li||Cu, Li||Li and Li||LFP cells used in this work are CR2032-type cells, which were assembled in Ar-filled glovebox. All the cells were assembled using 16 mm diameter Celgard 2325 separator, 12 mm diameter Li or Cu foil electrode and 30 μl electrolytes.

Material characterizations:

The solvent and solvation structure were characterized by Raman (JASCO microscope spectrometer, NRS-1000DT) with a laser excitation wavelength of 632.8 nm, and by FTIR (PerkinElmer, Spectrum Two LiTa). The electrodes were characterized using SEM (NRS-1000DT) and XPS (Thermo Scientific K-Alpha) after washed using EMC solvents. ^1H nuclear magnetic resonance (NMR) spectra were obtained using a coaxial nuclear magnetic resonance tube (BRUKER Nuclear Magnetic Resonance spectrometer), and 0.1 M LiFSI in H_2O/D_2O (98/2 v/v) is used as the internal axis standard sample. Lithium metal of 0.1 mA h was deposited on the Cu mesh at 0.1 mA for 1 h using Li||Cu cells. After cleaning with DME, it was stored, transferred and frozen in sample boxes protected by argon gas, and further characterized by cryo-transmission electron microscopy (Cryo-TEM).

Electrochemical measurements:

The Coulombic efficiency, charge discharge curve, and cycling performance of the cells was evaluated by the Land CT2001A battery test system and Neware System (MHWX-200). The ionic conductivity, Li^+ transference number and EIS plots of LHCEs was tested by CHI660E (Shanghai Chen Hua Instrument) or Biologic

(VSP-300). Distribution relaxation times (DRT) from EIS data were calculated by MatlabR2021 with a toolbox of DRT-TOOLS developed by the research group of Professor Francesco Ciucci1 (DRT-TOOLS is freely available under the GNU license from the following site: https://sites.google.com/site/drttools/).

The specific value was calculated by the following formula:

$$\sigma = \frac{d}{RS} \tag{1}$$

where σ is ionic conductivity, d is the thickness of separator, R is the resistance, and S is the aera of the SS.

The calculating formula of Li^+ transference number is shown as follow[1]:

$$t_{Li^+} = \frac{I_s(\Delta V - R_o I_o)}{I_o(\Delta V - R_s I_s)} \tag{2}$$

ΔV is the applied polarization potential (10 mV), I_o and I_s are the currents at initial and steady state, R_o and R_s stand for the resistance at initial and steady state.

Computational details:

The DFT and MD simulations were carried out on Dmol3 or Forcite module of Materials Studio of Accelrys Inc. A condensed−phase optimized molecular potentials for atomistic simulation studies (COMPASS II) force field was utilized. Temperature was set to be 298 K with Nose thermostat. All models have undergone sufficient duration of structural optimization to achieve the lowest energy. Then, the simulation models were equilibrated in canonical ensemble for 5 ns in the production stage for MD simulations. The simulation time was long enough to ensure reaching the equilibrium states of electrolytes.

Supporting Figures

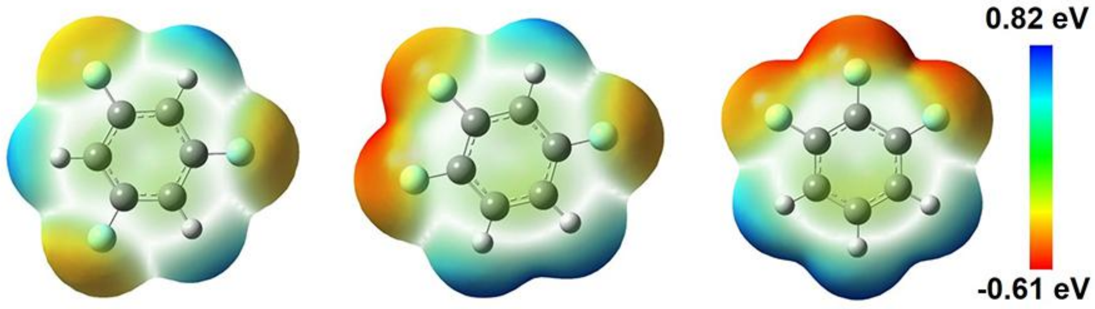

Figure S1. Electrostatic potential distribution diagrams of a-tFB, b-tFB, c-tFB.

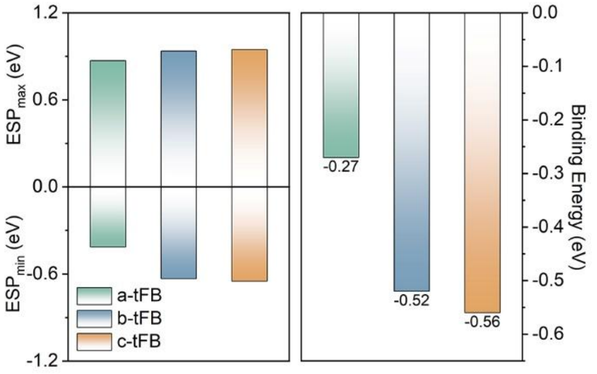

Figure S2. ESP_{max} and ESP_{min} values of three antisolvents and their binding energy with Li^+.

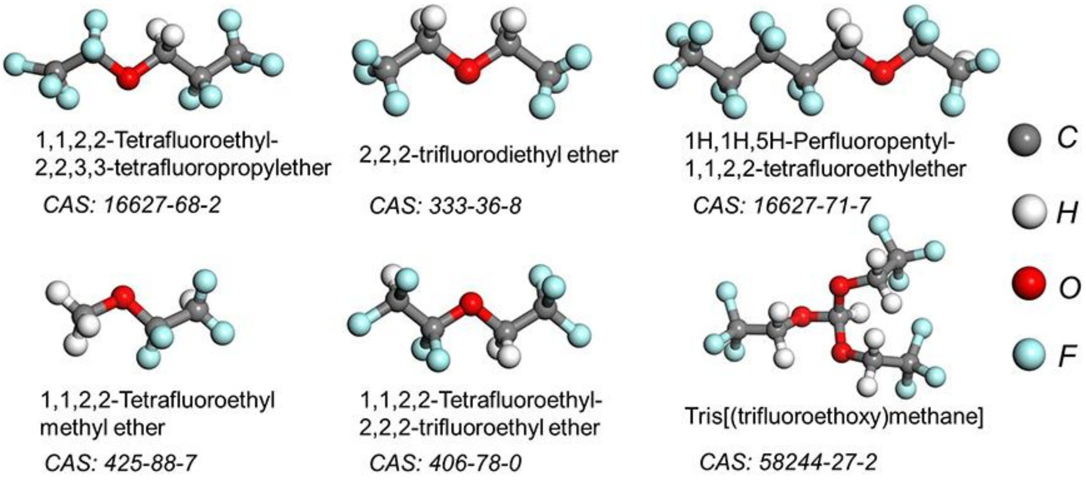

Figure S3. The ball stick model of common linear antisolvent molecules, whose coordination with other molecules/ions is influenced by steric hindrance dependent on molecular configuration.

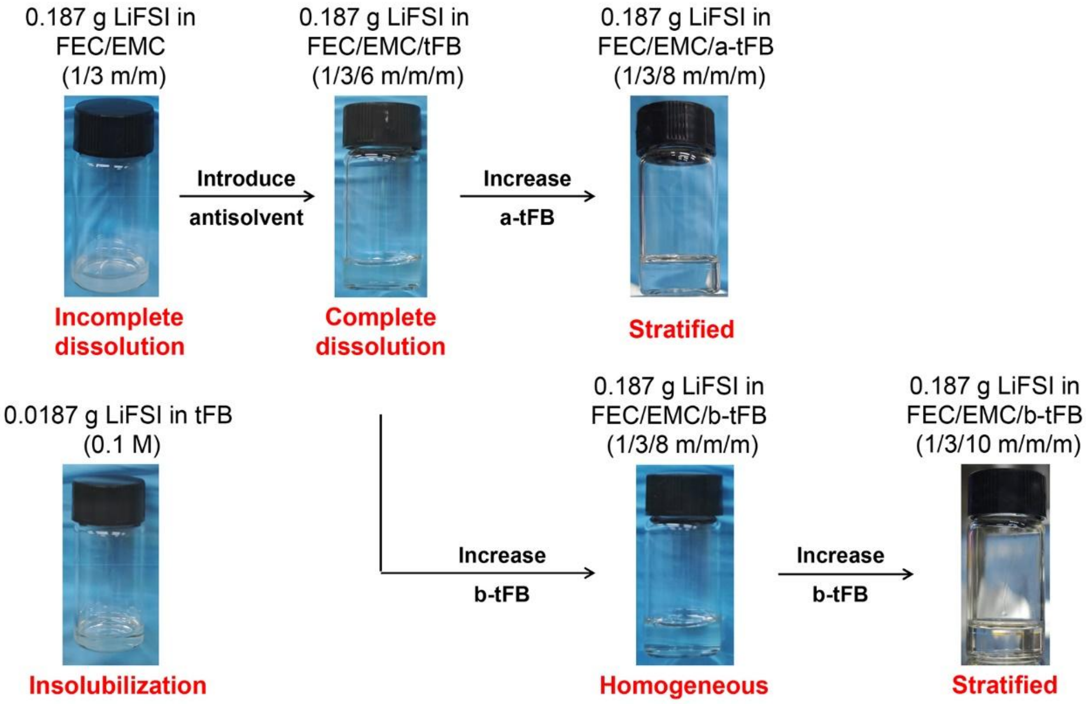

Figure S4. Experiments on the solubility of lithium salts in the main solvent and antisolvent, as well as the influence of the polarity and dosage of the antisolvent on the solubility of electrolytes.

Firstly, lithium salts (0.1M LiFSI) is insoluble in trifluorobenzene, which further confirms that trifluorobenzene can be used as an antisolvent for the LiFSI-based LHCE. Taking the target electrolyte as an example (FEC/EMC/tFB=1/3/6 mass ratio), when no antisolvent is introduced, the equivalent FEC/EMC mixed solvent cannot completely dissolve LiFSI. After further introduction of tFB, the lithium salt is completely dissolved. It is proved that the entropy increase effect of introducing antisolvent should increase the solubility of the LiFSI. However, when the content of the antisolvent was further increased and the mass proportion of the antisolvent was 2/3, the a-tFB electrolyte stratified, while the b/c-tFB electrolyte remained homogeneous. If the amount of the antisolvent is further increased, the latter will also stratify. The stronger the polarity of the antisolvent is, the better its miscibility with the main solvent will be.

It is important to note that while trifluorobenzene constitutes approximately 50% of the total volume of the electrolyte, its relatively low flash point of 62℃ renders the electrolyte flammable, thereby presenting a significant safety risk.

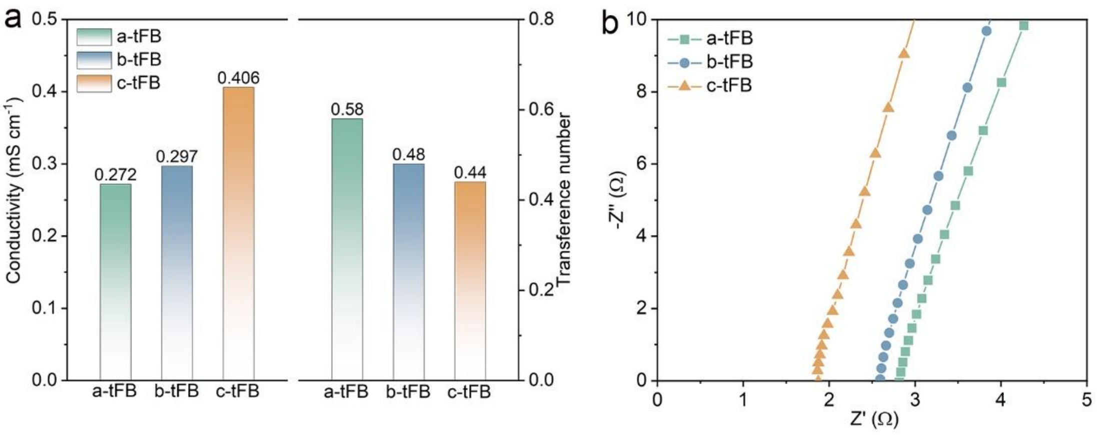

Figure S5. (a) The conductivity and Li^+ transference number of LHCEs with different antisolvents. (b) The EIS spectrum of SS||SS cells using different LHCEs, which is used to calculate ion conductivity.

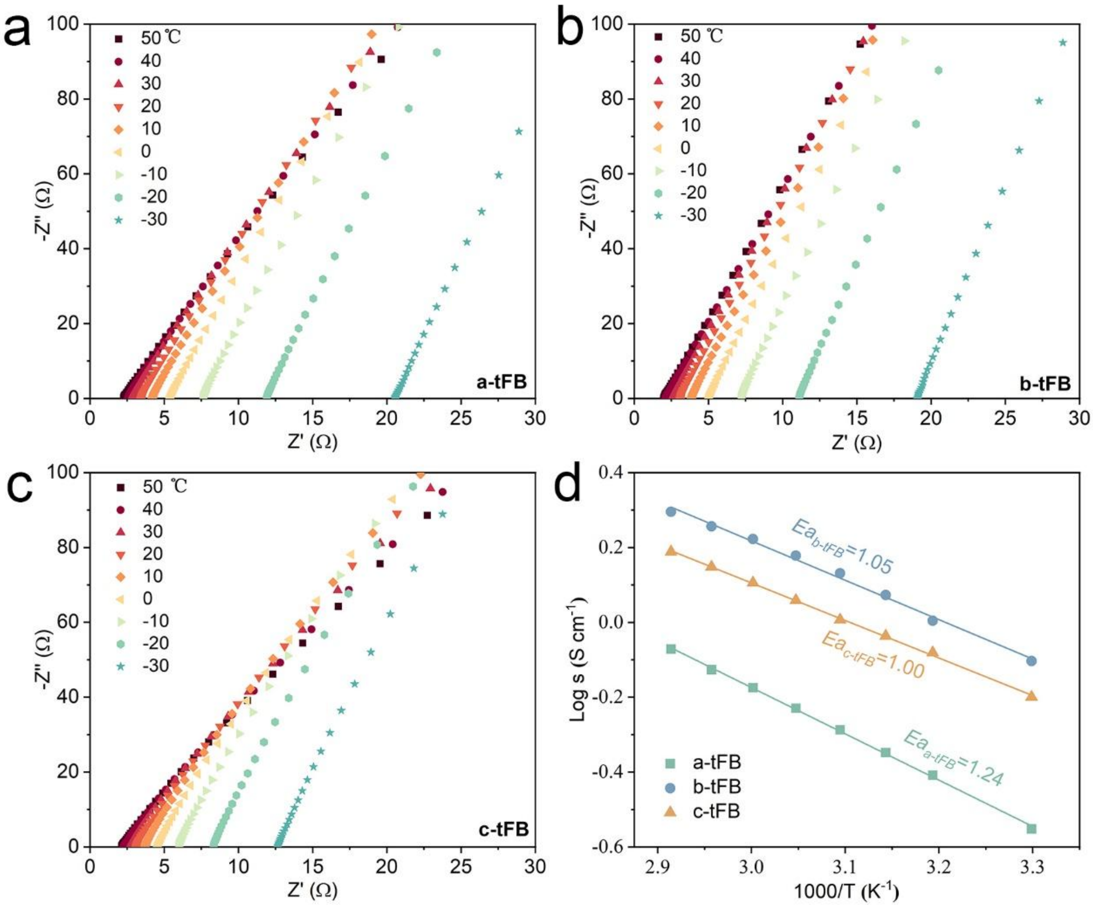

Figure S6. (a-c) Nyquist plots for the symmetric cells consisting of stainless-steel electrodes in LHCEs with different antisolvents at different temperatures ranging from -30 to 50 ℃. (d) Arrhenius plot for the resistance of Li^+ migration through LHCEs with different antisolvents.

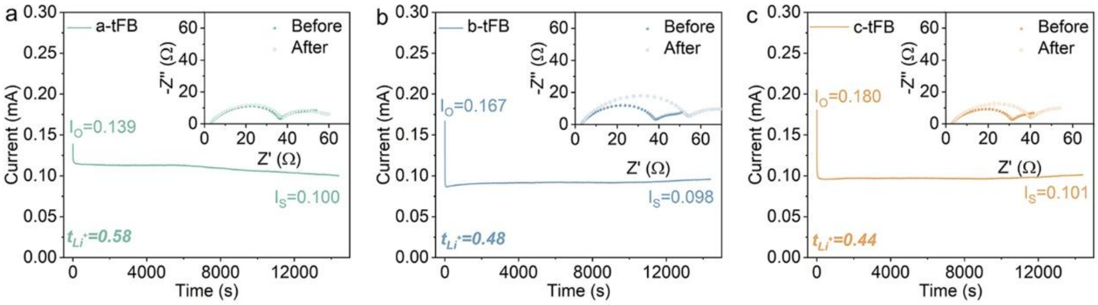

Figure S7. (a-c) CA polarization curves with an applied voltage of 10 mV for the symmetric cells consisting of Li electrodes in LHCEs with (a) a-tFB, (b) b-tFB and (c) c-tFB, respectively. Insets showing the corresponding Nyquist plots before and after CA polarization.

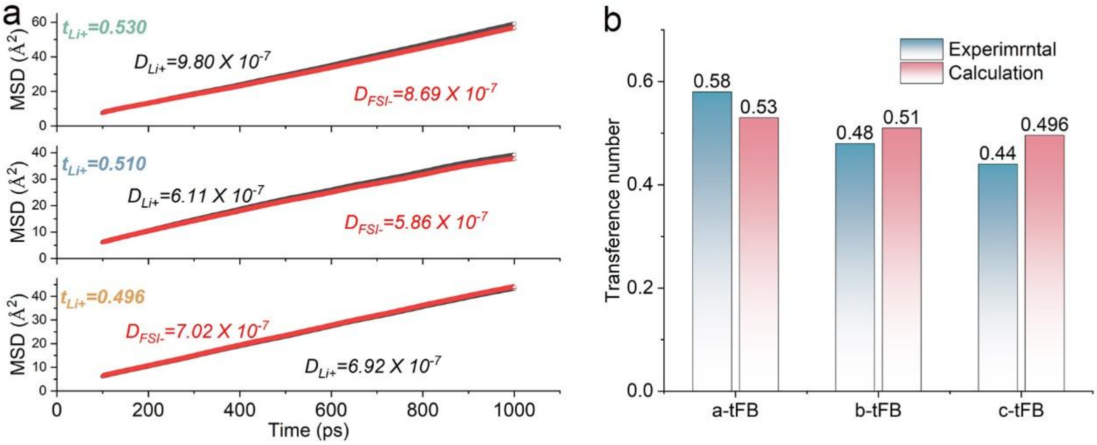

Figure S8. (a) The Li^+ transference number of LHCEs with different antisolvents, which are obtained by MD calculation. (b) Comparison of experimental and calculated Li^+ transference number.

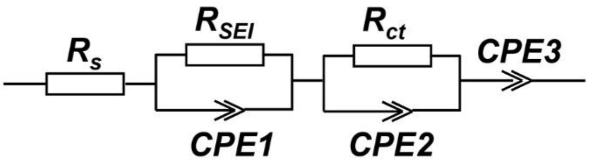

Figure S9. The equivalent circuit model for EIS date fitting.

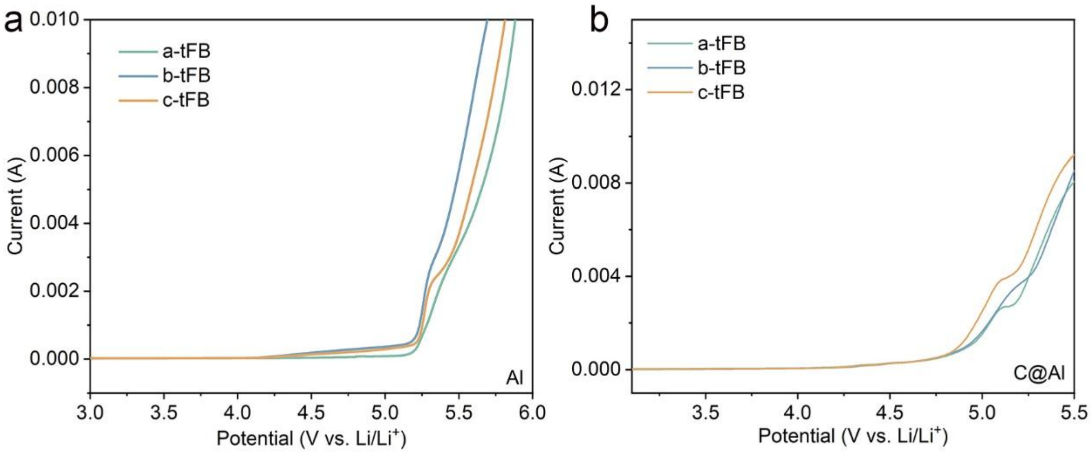

Figure S10. LSV curves of Li||Al cells (a) and Li||C@Al cells (b) using LHCEs with different antisolvents.

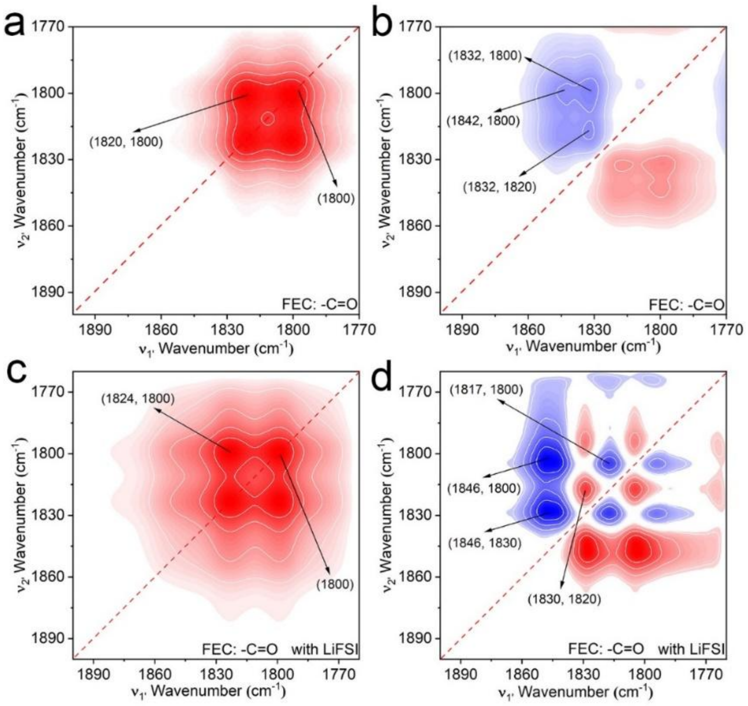

Figure S11. (a) Synchronous and (b) asynchronous 2D IR correlation spectra for the FEC mixed with different antisolvents in 1900-1770 cm^{-1} region. (c) Synchronous and (d) asynchronous 2D IR correlation spectra for the FEC in LHCEs with different antisolvents in 1900-1755 cm^{-1} region.

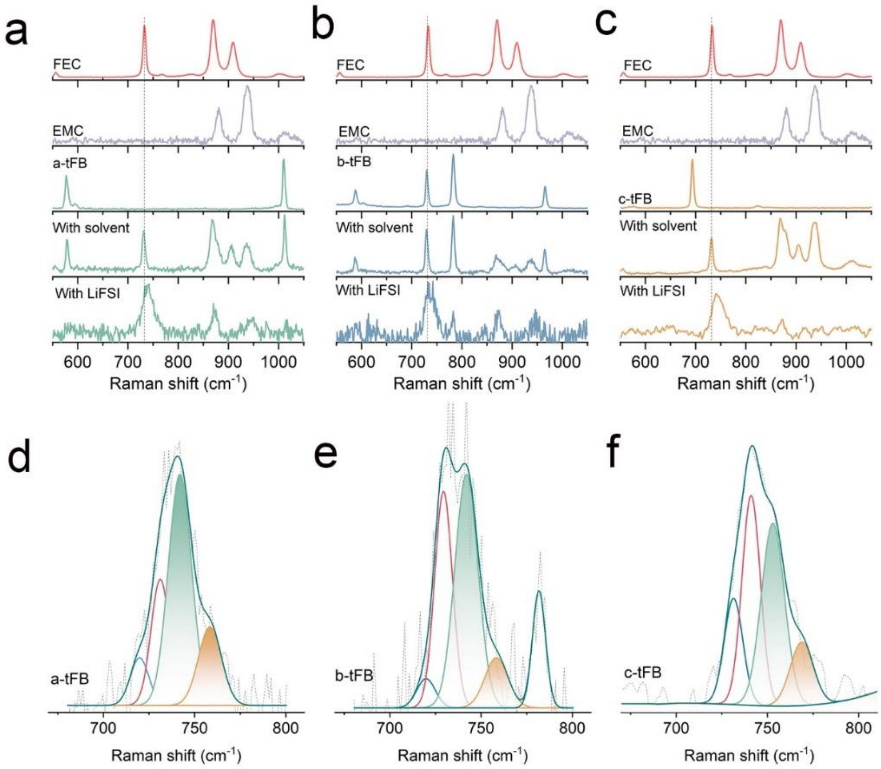

Figure S12. (a-c) Raman spectra for FEC, EMC, pure antisolvents, mixture of antisolvent and main solvent, and LHCEs with different antisolvents. Raman spectra for FSI^- anions in LHCEs with a-tFB (d), b-tFB (e) and c-tFB (f) as antisolvent, respectively.

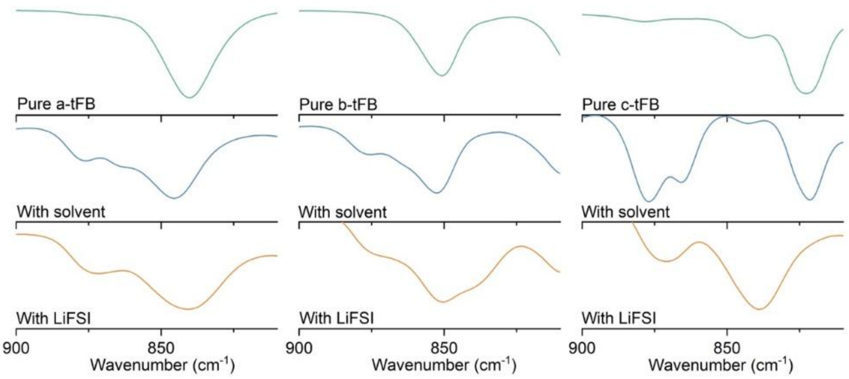

Figure S13. Aromatic hydrogen out-of-plane bending-ring skeleton vibration characteristic peak in FT-IR spectra of different pure antisolvents, antisolvents mixed with main solvents and LHCEs.

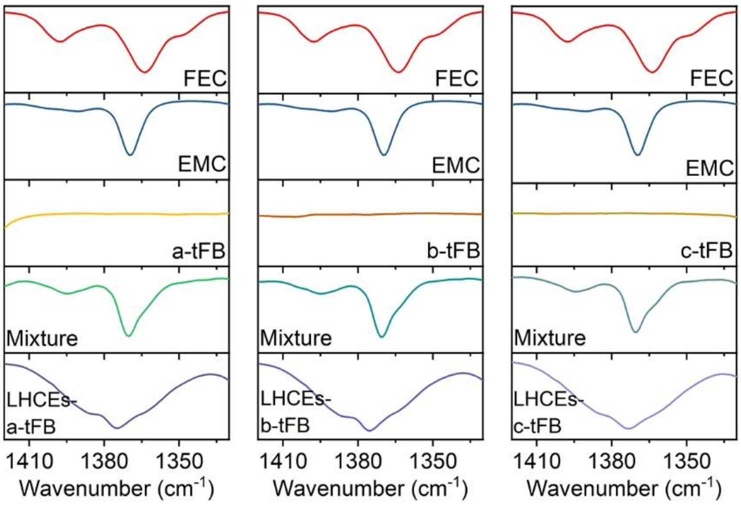

Figure S14. FT-IR spectra of FEC, EMC, trifluorobenzene and their mixture in the wavenumber ramge of 1420-1330 cm^{-1}.

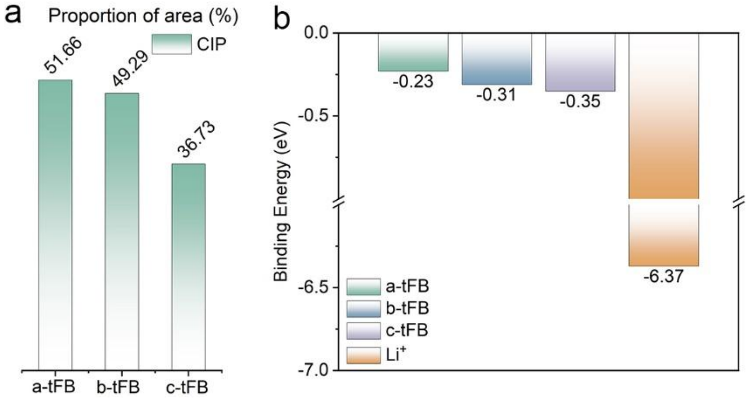

Figure S15. (a) Area proportion of CIP and AGG solvation structure in LHCEs with a-tFB, b-tFB and c-tFB as antisolvent. (b) The binding energy between FSI- with a-tFB, b-tFB, c-tFB and Li^+.

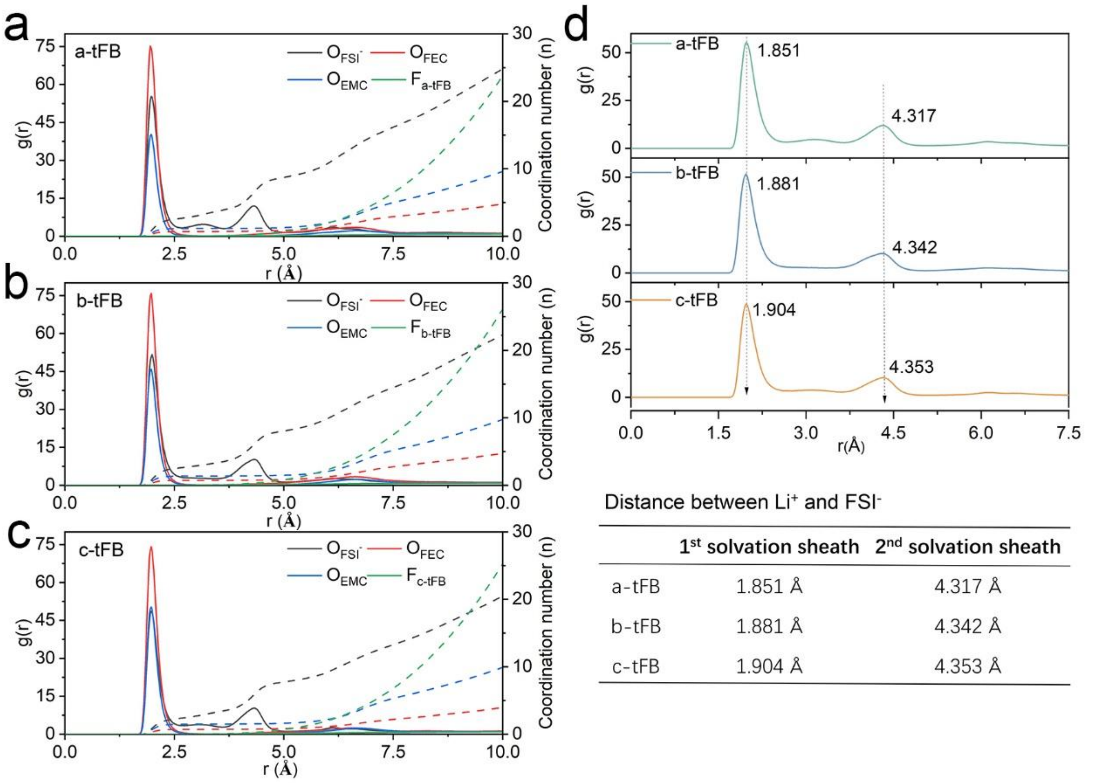

	1st solvation sheath	2nd solvation sheath
a-tFB	1.851 Å	4.317 Å
b-tFB	1.881 Å	4.342 Å
c-tFB	1.904 Å	4.353 Å

Figure S16. Radical distribution functions (RDFs, g(r), solid) and cumulative distribution functions (CDFs, n(r), dashed line) of oxygen around Li^+ as a function of distance (r) from MD simulations in LHCEs with a-tFB (a), b-tFB (b) and c-tFB (c), respectively. (d) The RDF comparison diagram of FSI^- anions around Li^+, and the distance between Li^+ and FSI^- in 1st and 2nd solvation sheath.

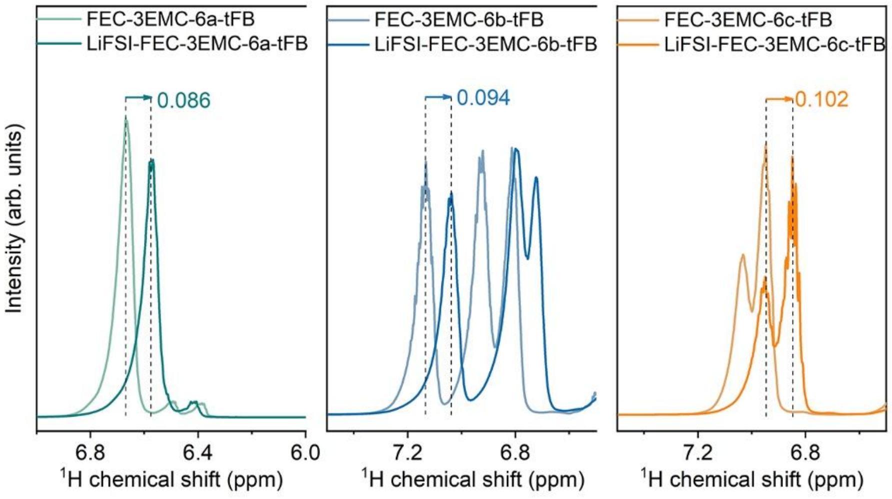

Figure S17. ^1H NMR spectra of mixture solvent containing a/b/c-tFB with/without LiFSI.

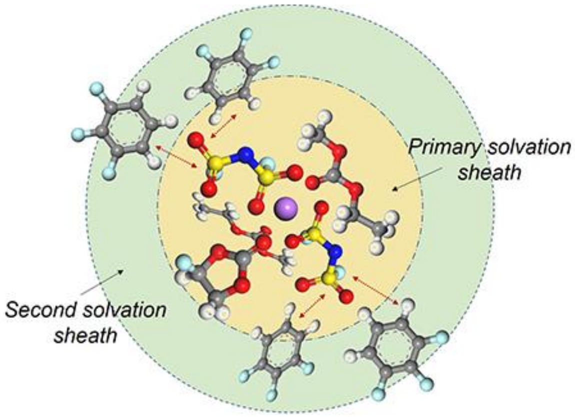

Figure S18. Schematic diagram of various interactions in the micellar solvation structure of LHCEs.

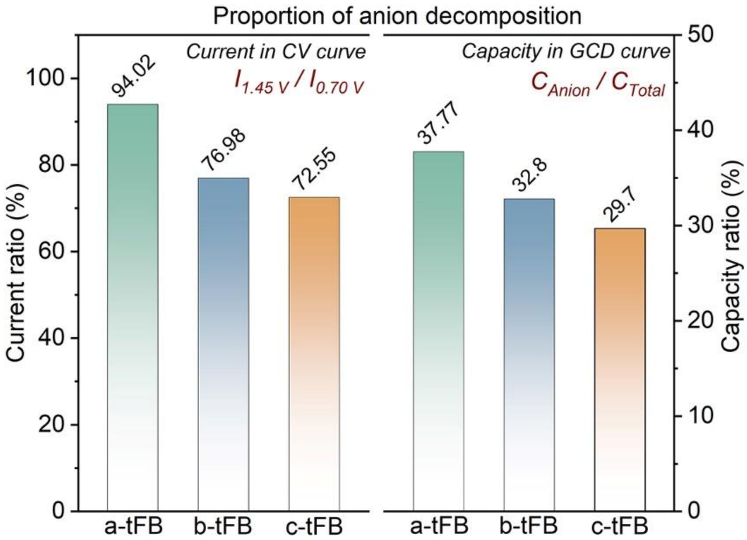

Figure S19. The peak current ratio of the reduction peak in the 1.45 V and 0.70 V regions of the CV curves of LHCEs with different antisolvents. The proportion of anion decomposition capacity in the total discharge capacity in different LHCEs.

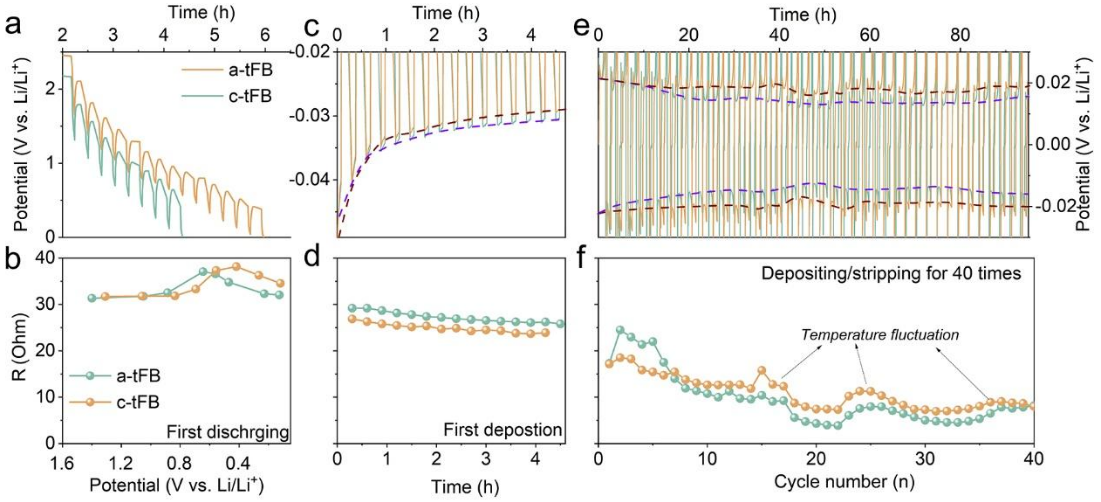

Figure S20. The potential profiles and their real-time testing interface impedance values of the three stages of SEI film formation in Li||Cu cells using electrolyte with a-tFB and c-tFB as antisolvent, respectively, including the stage-1 with potential greater than 0 V vs. Li/Li^+ (a, b), stage-2 corresponding to the first Li deposition process (c, d) and stage-3 corresponding to the first 40 times depositing/stripping (e, f).

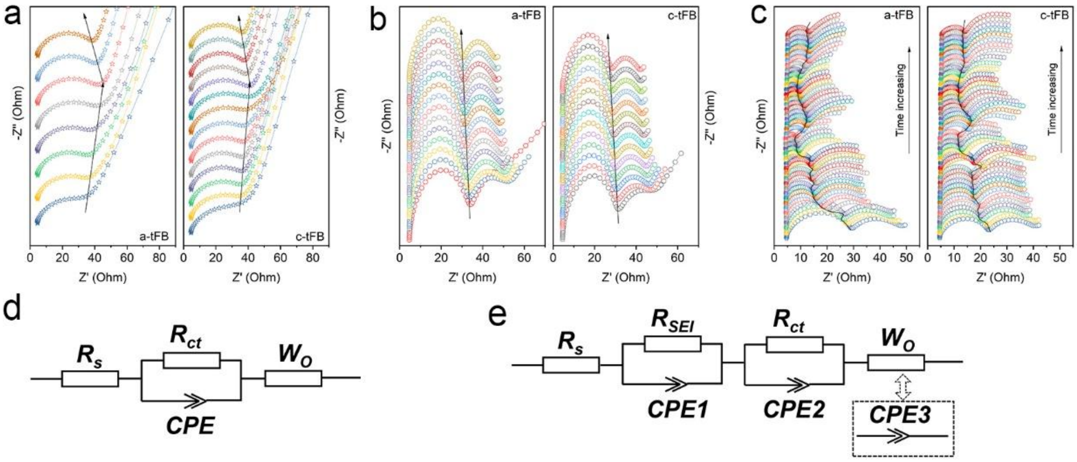

Figure S21. (a-c) In-situ Nyquist plots for the three stages of SEI film formation in Li||Cu cells using electrolyte with a-tFB and c-tFB as antisolvent, respectively. The equivalent circuit model for EIS date fitting in Figure S19a (d), or Figure S19b-c (e). Flexibly adjust the specific mode of its equivalent circuit according to the differences in the low-frequency region.

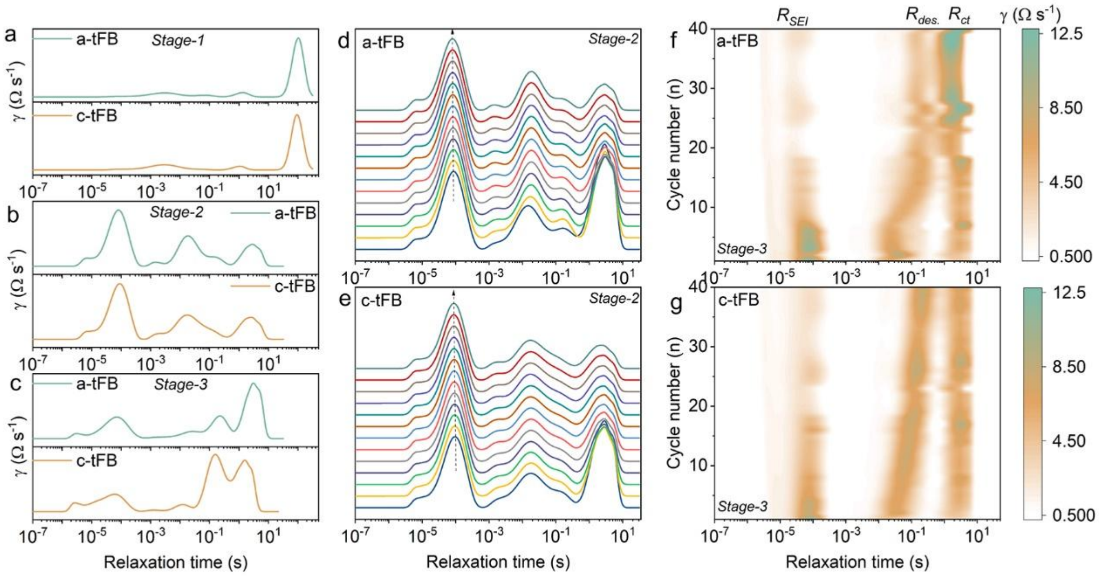

Figure S22. (a) The DRT curves in *stage-1* (a), *stage-2* (b) and *stage-3* (c) during the SEI film formation process. In-situ DRT curves for the first discharging of Li||Cu cell in a-tFB (d) and c-tFB (e) at the potential range of low than 0 V vs. Li/Li^{+}. In-situ DRT mappings for the first 40 cycles of Li||Cu cells in a-tFB (f) and c-tFB (g).

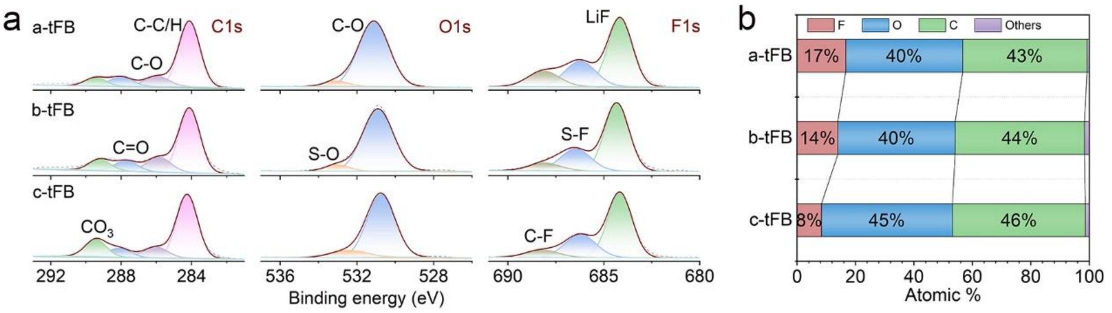

Figure S23. (a) C1s, O1s and F1s spectra of Li foil after 20 cycles of Li deposition/stripping in different LHCEs. (b) Atomic ratio in SEI film formed in different LHCEs.

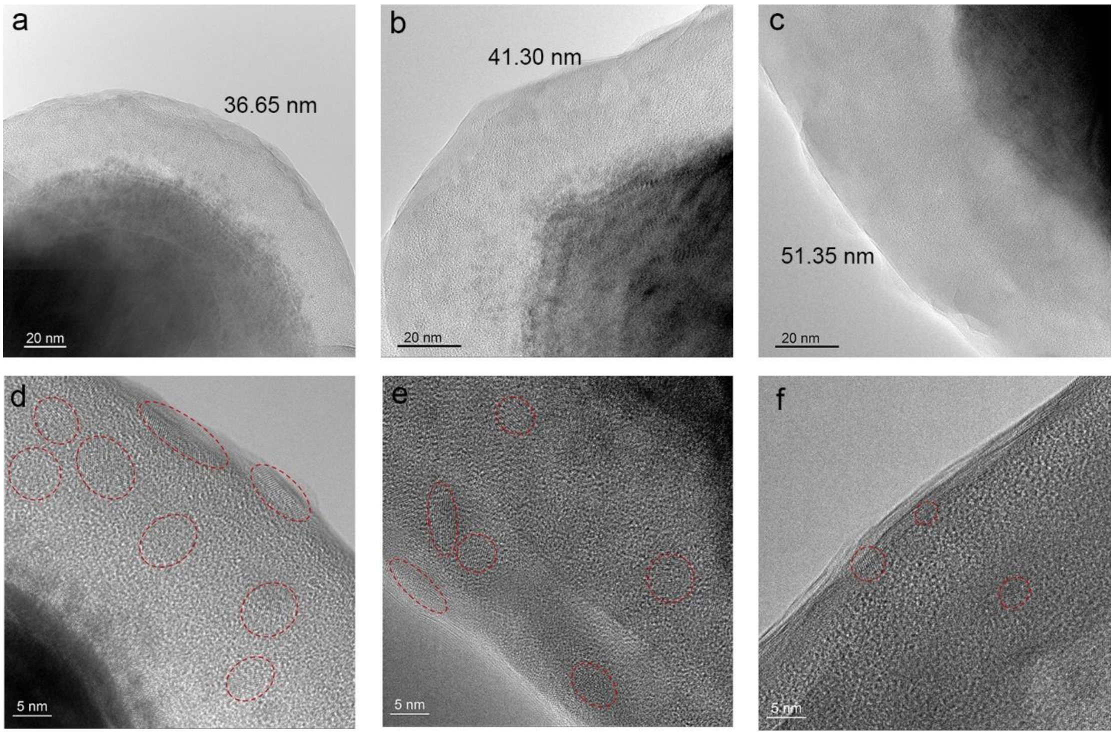

Figure S24. Cryo-TEM images and high-resolution images of SEI films on Li metal anode formed in a-tFB (a,d), b-tFB (b,e) and c-tFB (c,f), respectively. The red ellipses marked the lattice fringe images specific to the inorganic components in the SEI film.

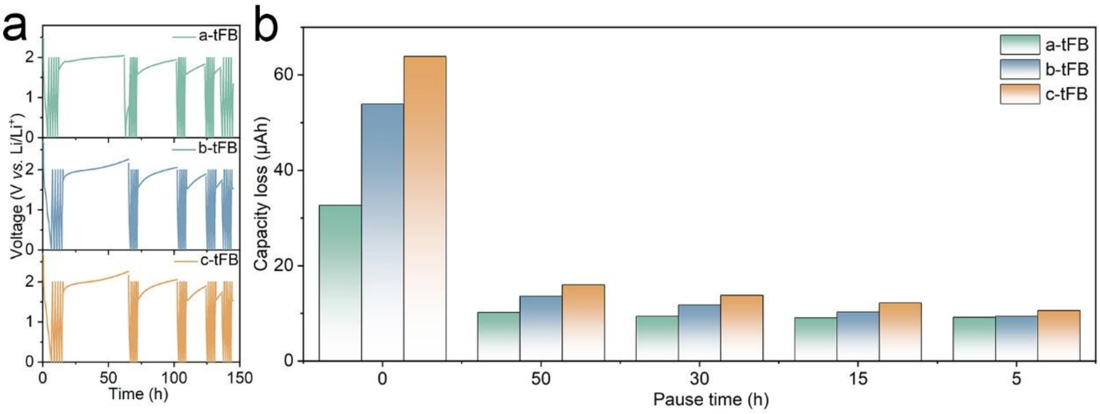

Figure S25. SEI dissolution analysis by capacity loss of Cu||Li cells in three electrolytes. (a)The voltage profiles at current of 10 μA and (b) the capacity loss for different pause times in three electrolytes.

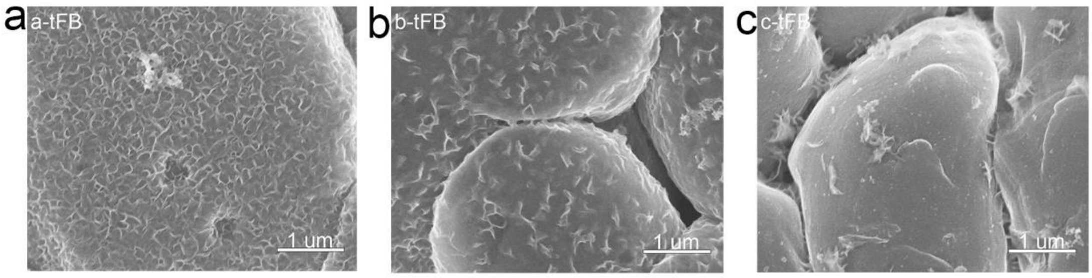

Figure S26. SEM images of Li anode deposited in a-tFB (a), b-tFB (b) and c-tFB (c), respectively.

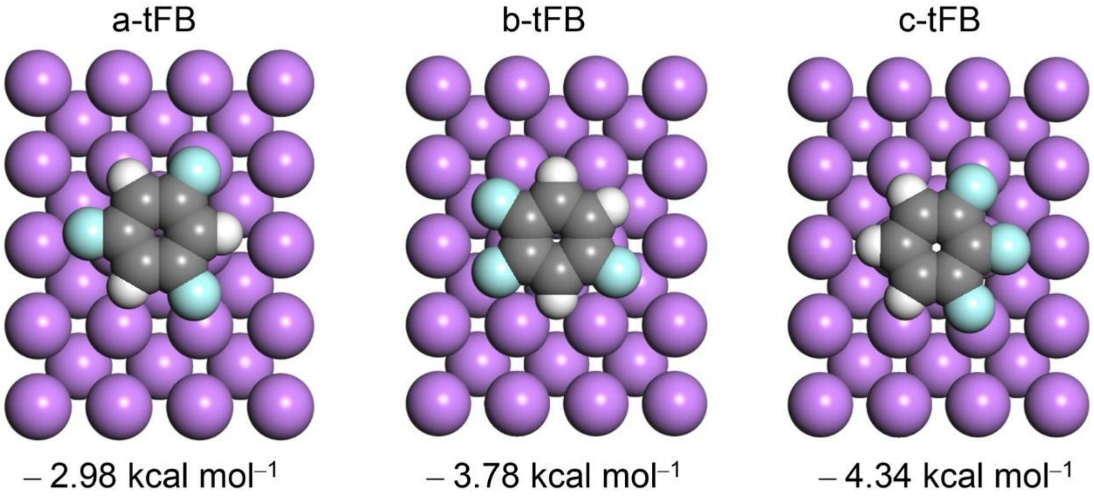

Figure S27. The adsorption energy of different antisolvent molecules on the Li metal (002) crystal plane in parallel configurations.

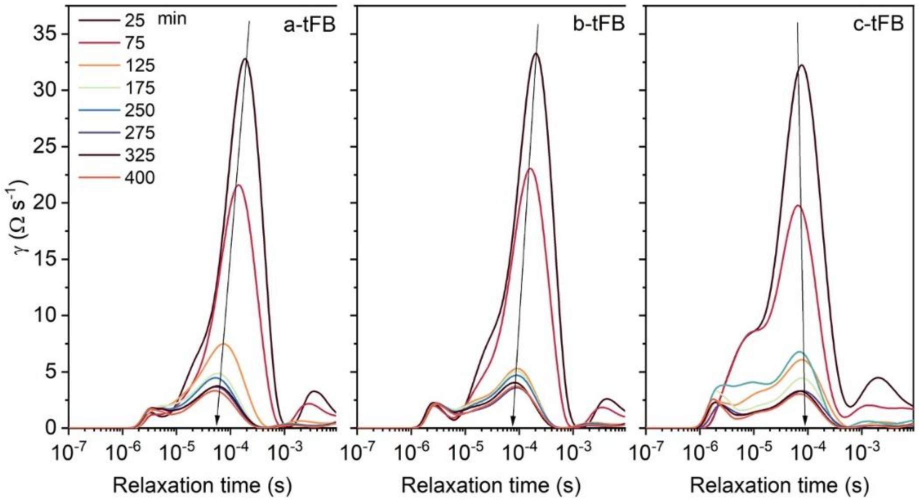

Figure S28. In-situ DRT curves at first 400 min for first discharging of Li||Li cells using LHCEs with different antisolvents.

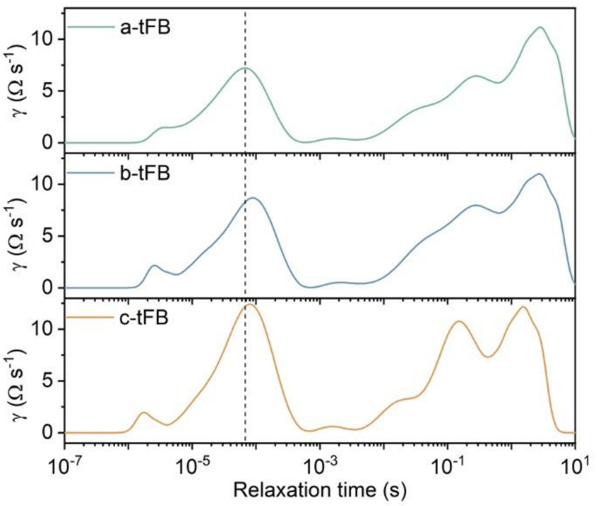

Figure S29. DRT curves after SEI film formation in LHCEs with different antisolvents.

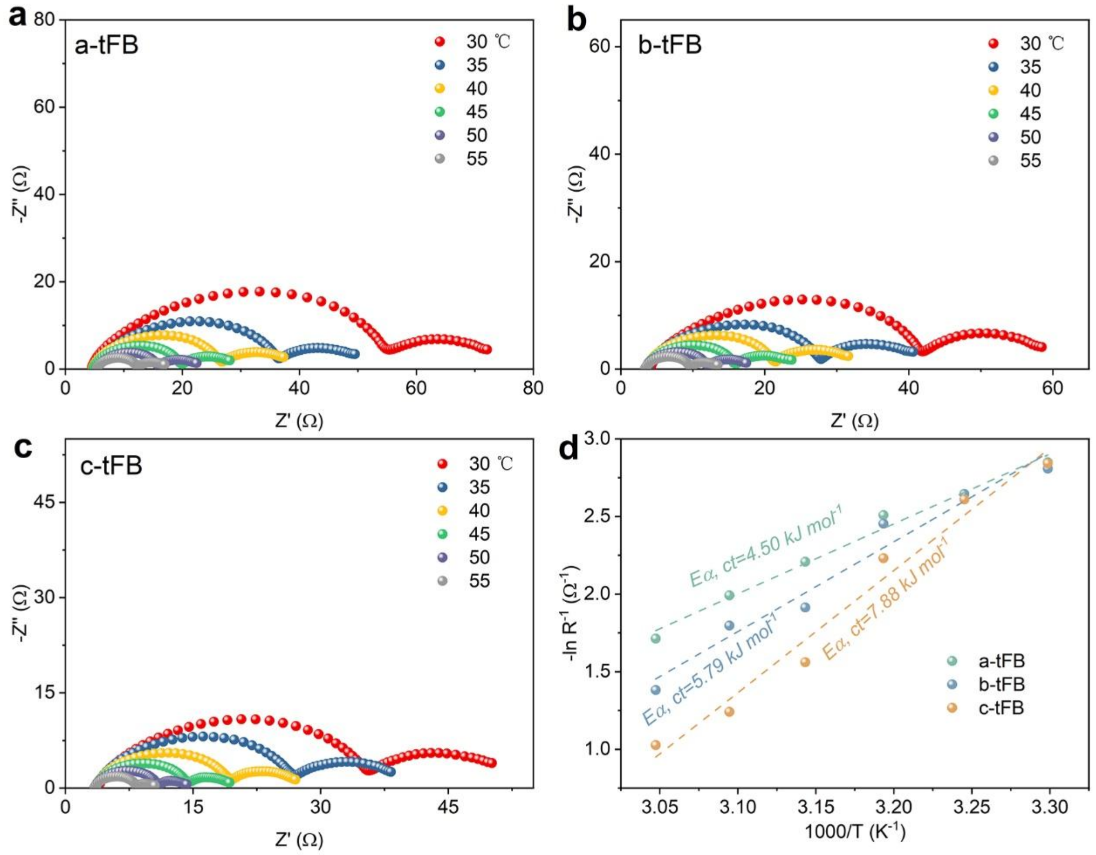

Figure S30. (a-c) Nyquist plots for the Li||Li cells using electrolyte with a-tFB (a), b-tFB (b) and c-tFB (c) as antisolvent at different temperature, respectively. (d) Arrhenius behavior of the Rct in (a-c), corresponding to the lithium ions desolvation process.

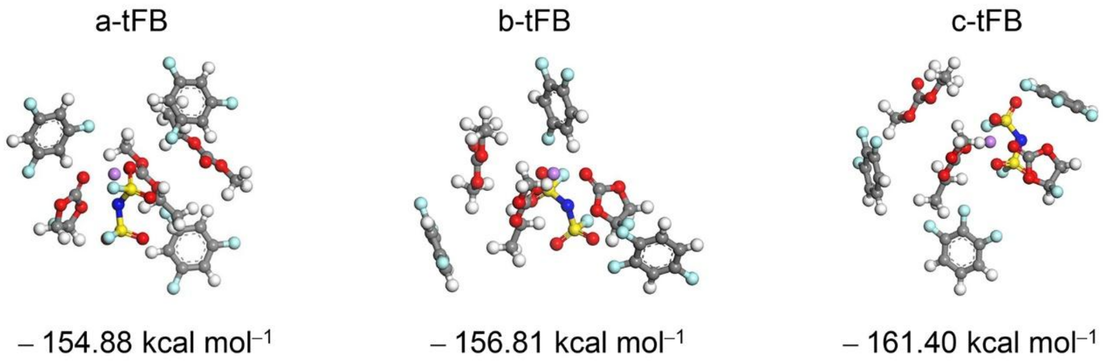

Figure S31. The desolvation energy of three kinds of electrolytes with different antisolvents, which are calculated by first principles.

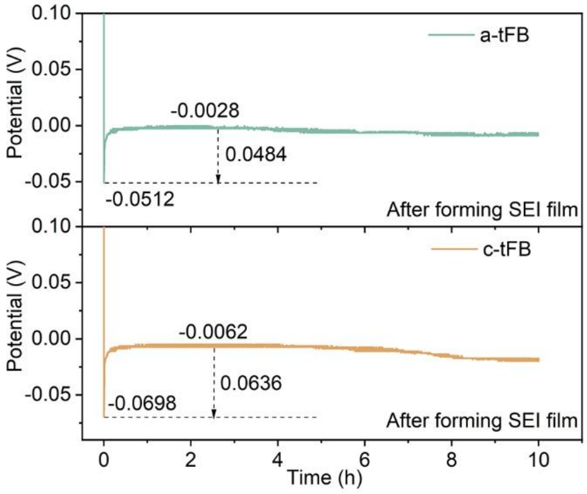

Figure S32. The voltage profiles of lithium deposition on Cu foils after forming stable SEI films in a-tFB and c-tFB, respectively. The deposition overpotential and nucleation overpotential are clearly marked in the figure.

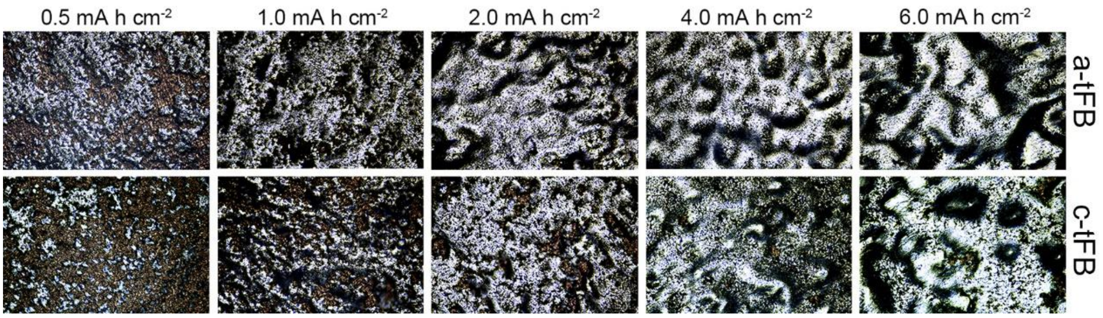

Figure S33. Optical photographs of lithium deposition morphology on Cu foil surface under different deposition capacities in different electrolytes (Picture size: 1300*850 μm).

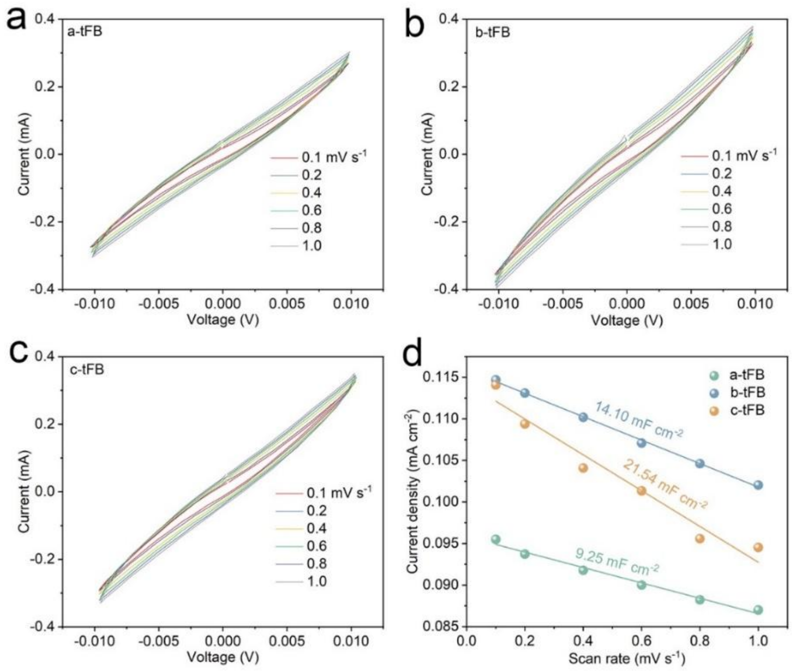

Figure S34. (a-c) CV curves of Li||Li cells with different electrolytes in a voltage range of -10 mV and 10 mV after cycling 10 cycles at 1.0 mA cm-2. (d) Plots of capacitive currents versus scan rates for different LHCEs.

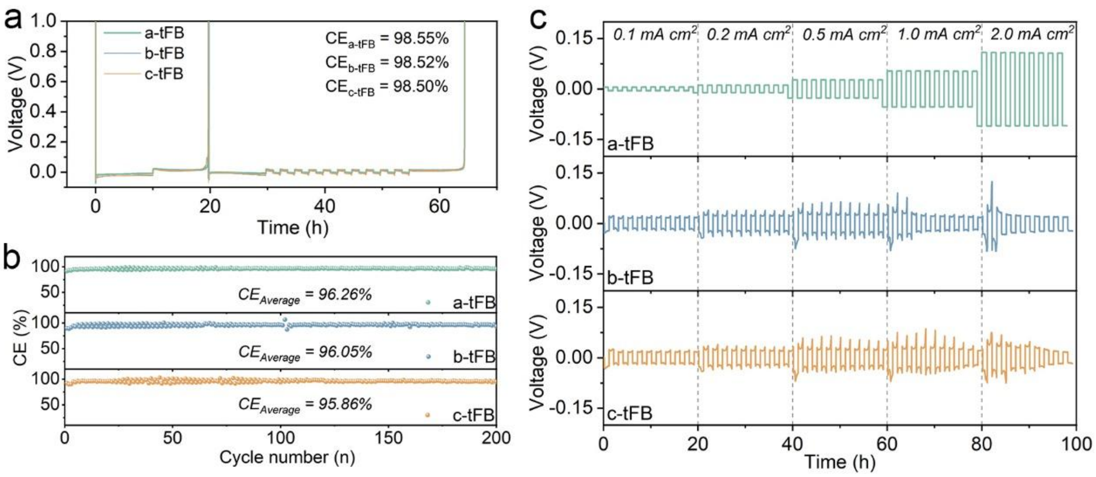

Figure S35. (a) CEs of Li||Cu cells with different LHCEs by the modified Aurbach method. (b) CEs of Li||Cu cells at a current density of 0.5 mA cm^{-2} for 0.5 mAh cm^{-2}. (c) Rate performance of Li||Li cells using different LHCEs at different current densities.

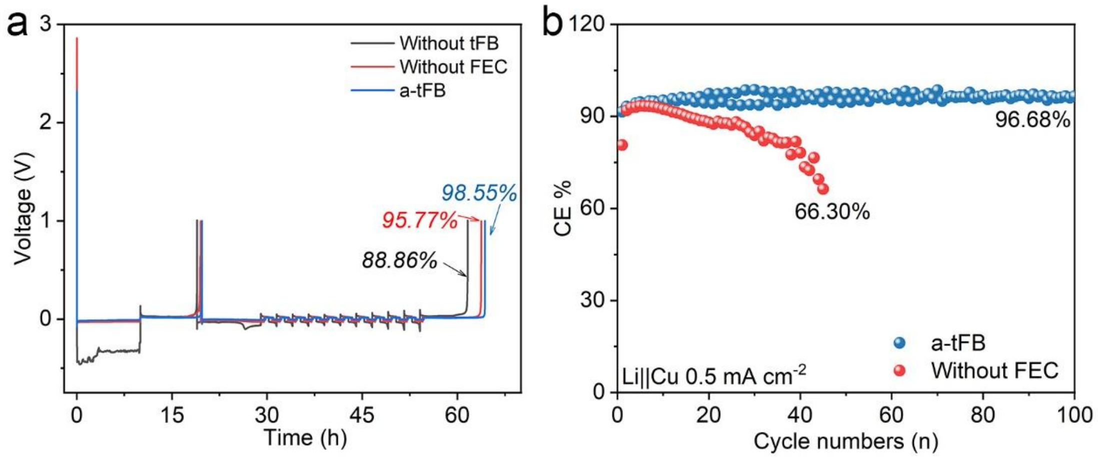

Figure S36. CEs of Li||Cu cells using target electrolytes (a-tFB), electrolytes without trifluorobenzene, or without FEC by improved Aurbach method (a) or long cycling tests at 0.5 mA cm^{-2} for 0.5 mA h cm^{-2} (b).

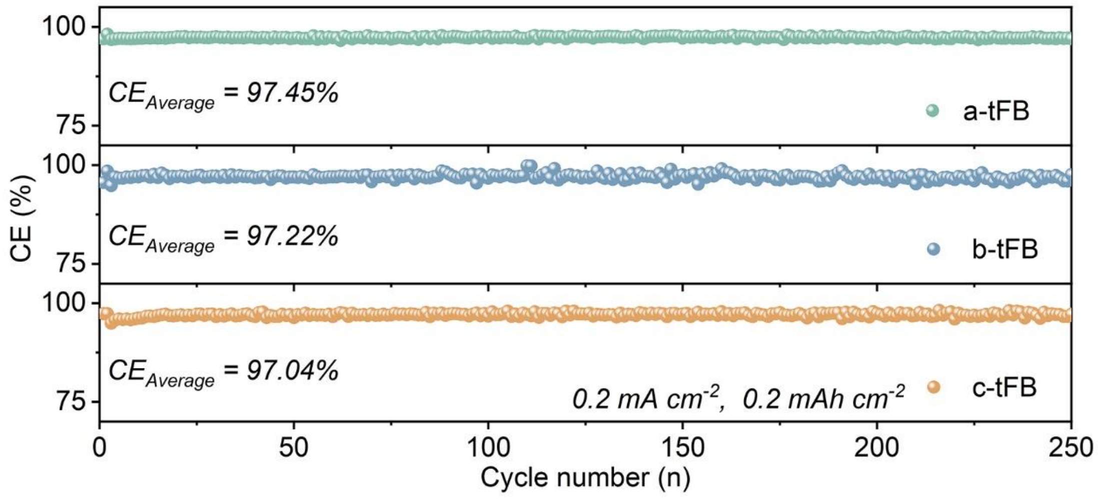

Figure S37. CEs of Li||Cu cells at a current densigu of 0.2 mA cm^{-2} for 0.2 mAh cm^{-2}.

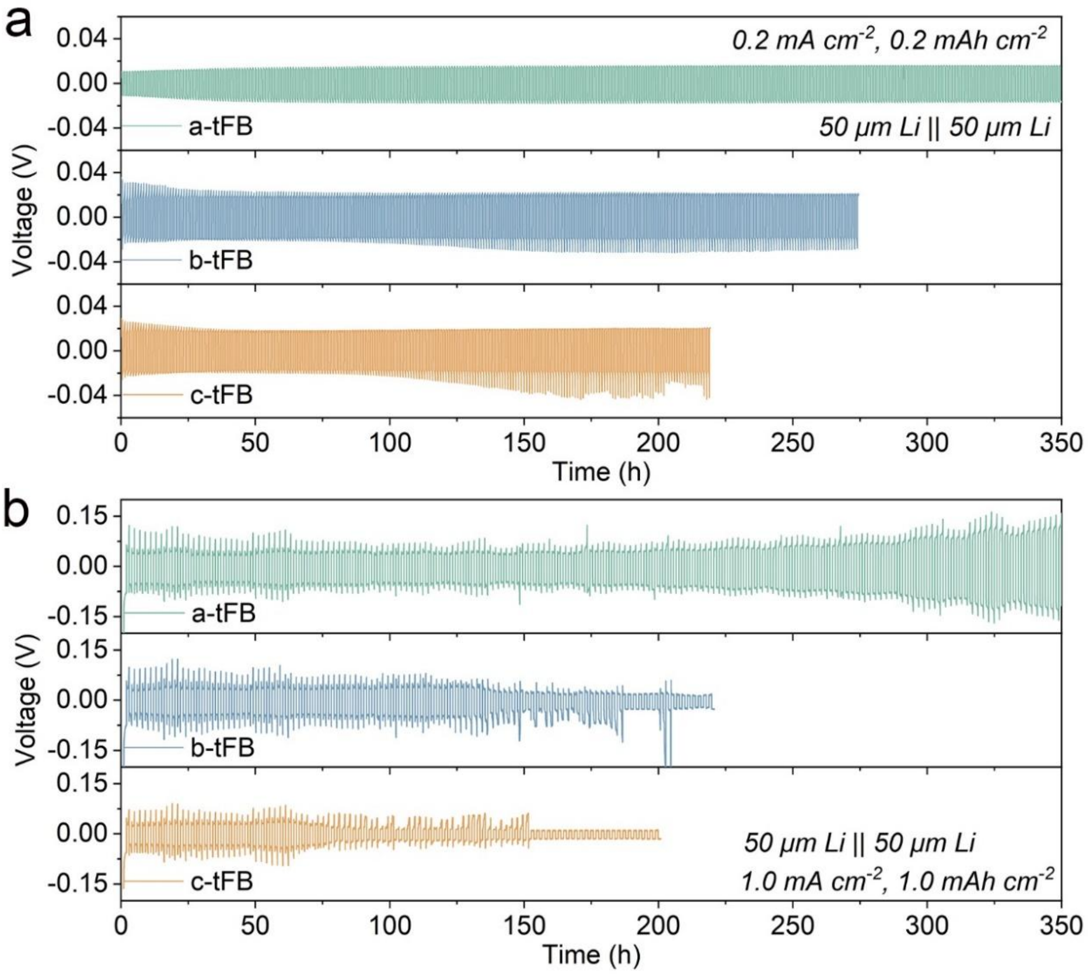

Figure S38. Cycle performance of Li||Li cells with limited Li (50 μm) in different LHCEs at current density of 0.2 mA cm^{-2} (a) and 1.0 mA cm^{-2} (b).

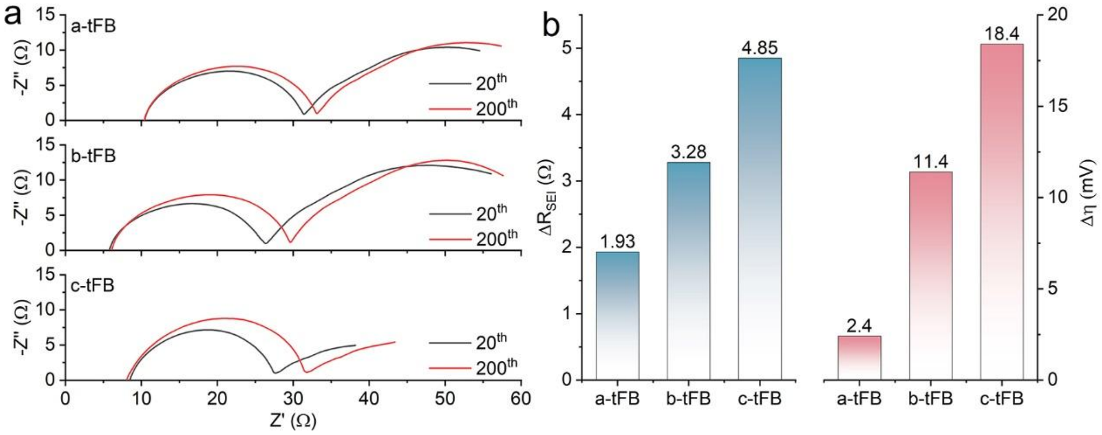

Figure S39. Typical voltage profiles and EIS plots of Li||Li cells using different LHCEs at 10th cycle and 200th cycle.

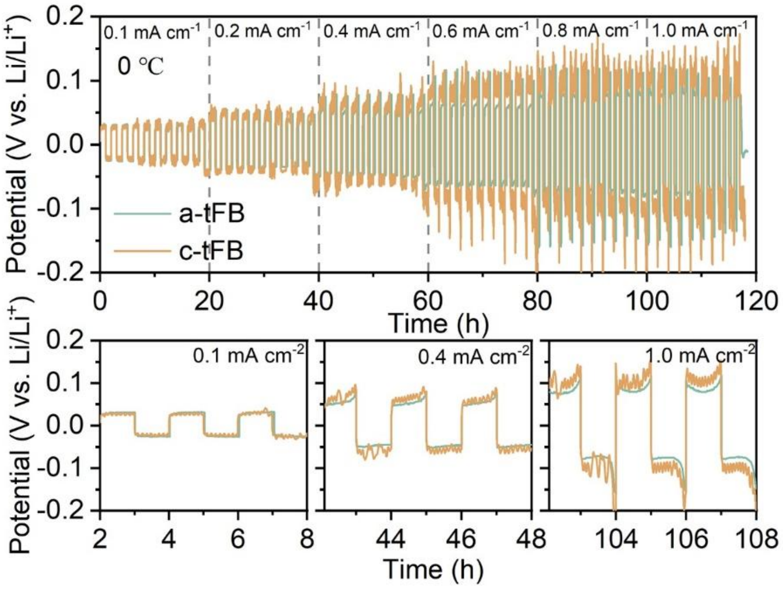

Figure S40. Rate performance and typical voltage profiles of Li||Li cells using different LHCEs at different current densities at 0 ℃.

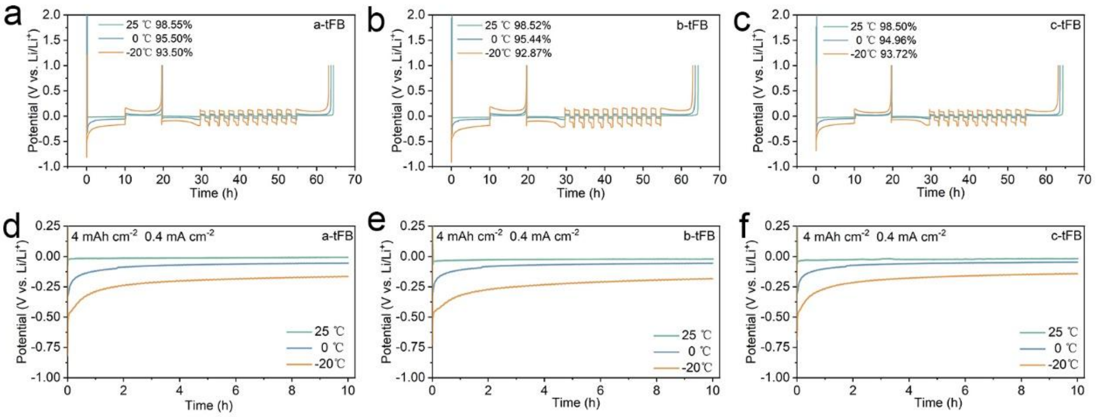

Figure S41. (a) CEs of Li||Cu cells at different temperatures by the modified Aurbach method in a-tFB (a), b-tFB (b) and c-tFB (c), respectively. The potential profiles of the first Li deposition process in Li||Cu cells at different temperatures using a-tFB (d), b-tFB (e) and c-tFB (f), respectively.

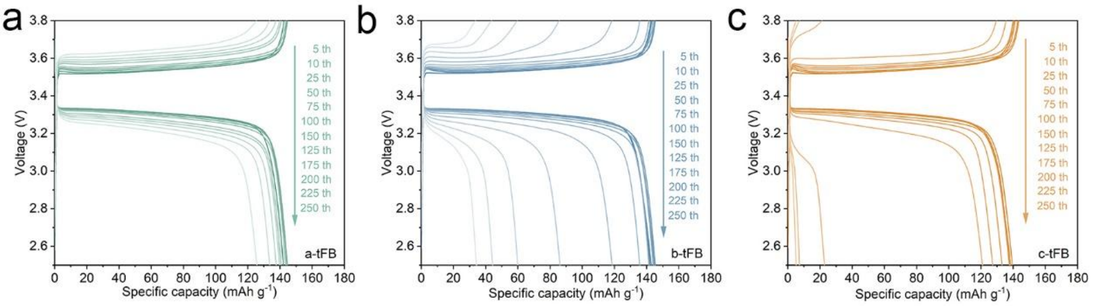

Figure S42. The charge-discharge curves of Li||LFP cells at different cycles using a-tFB (a), b-tFB (b) and c-tFB (c), respectively.

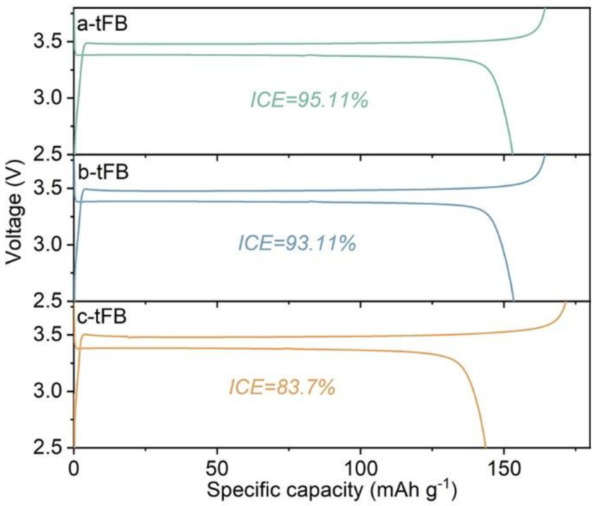

Figure S43. The first charge-discharge curves of Li||LFP cells at different cycles using different electrolytes.

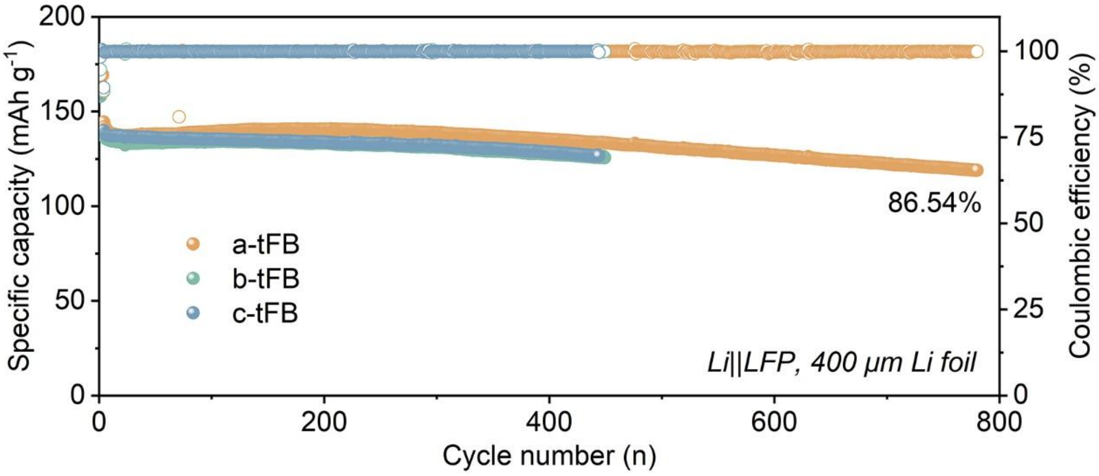

Figure S44. The cycling performance of Li||LFP full cells with 400 μm Li anode using LHCEs with different antisolvents.

References:

[1] P.G. Bruce, J. Evans and C.A. Vincent, ***Solid State Ionics***, 1988, 28, 918-922.

Table S1. The property parameters of three antisolvents.

	**Melting point (℃)**	**ESP_{max} (eV)**	**ESP_{min} (eV)**	**Density (g ml^{-1})**
**a-tFB**	75	0.871	-0.414	1.277
**b-tFB**	88	0.937	-0.632	1.264
**c-tFB**	95	0.947	-0.650	1.28

Table S2. The average CES of Li||Cu cells using ester-based electrolytes.

Electrolytes	Current density	Capacity	Cycling performance	Ref.
1 M LiFSI in FEC/EMC/a-tFB	0.5 mA cm^{-2}	0.5 mA h cm^{-2}	Average CE ~96.26% 200 cycles	This work
	0.2 mA cm^{-2}	0.2 mA h cm^{-2}	Average CE ~97.45% 200 cycles	
1 M $LiPF_6$ in DMDOHD	0.5 mA cm^{-2}	1 mA h cm^{-2}	Average CE ~92% > 250 cycles	Nat. Commun. 2024, 15, 536
	1.0 mA cm^{-2}		Average CE ~87% > 150 cycles	
1 M $LiPF_6$ in EC	0.5 mA cm^{-2}	1 mA h cm^{-2}	CE ~40% Dead at ~100 cycles	ACS Energy Lett. 2021, 6, 69-78.
1 M $LiPF_6$ in DMC	0.5 mA cm^{-2}	1 mA h cm^{-2}	CE ~20% ~100 cycles	
1 M $LiPF_6$ in DEC	0.5 mA cm^{-2}	1 mA h cm^{-2}	CE ~5% Dead at ~20 cycles	
1 M $LiPF_6$ in EMC	0.5 mA cm^{-2}	1 mA h cm^{-2}	CE ~1.3% Dead at ~50 cycles	
1 M $LiPF_6$ in PC	0.2 mA cm^{-2}	0.4 mA h cm^{-2}	CE ~73.2% 10 cycles	ACS Energy Lett. 2018, 3, 14-19.
1 M $LiPF_6$ in EC/DMC (1/1 vol.)	1 mA cm^{-2}	1 mA h cm^{-2}	CE ~85% 70 cycles	Adv. Energy Mater. 2020, 10, 1903568.
1 M $LiPF_6$ in EC/DEC	1 mA cm^{-2}	1 mA h cm^{-2}	CE ~88% Quickly fails within 20 cycles	Angew. Chem. Int. Ed. 2020,59, 14935-14941.
1 M $LiPF_6$ in EC/EMC	0.5 mA cm^{-2}	0.5 mA h cm^{-2}	CE ~62.7% Dead at 170 cycles	Nano Energy 2020, 74, 104860.
1 M $LiPF_6$ in FEC/EMC	0.5 mA cm^{-2}	0.5 mA h cm^{-2}	CE ~83.4% Dead at ~300 cycles	
1 M $LiPF_6$ in FEC/DMC (1/4 vol.)	1 mA cm^{-2}	1 mA h cm^{-2}	CE ~33% Dead at ~41 cycles	Angew. Chem. Int. Ed. 2021, 60, 3661-3671.
1 M $LiPF_6$ in EC/DEC (1/1 vol.)	0.25 mA cm^{-2}	1 mA h cm^{-2}	CE < 80% After 50 cycles	ACS Appl. Mater. Interfaces 2019, 11,

				6118-6125
	1 mA cm^{-2}	2 mA h cm^{-2}	CE < 10% Within 30 cycles	J. Phys. Chem. C 2018, 122, 21462-21467
1 M $LiPF_6$ in EC/DEC (1/1 vol.) + 2 wt.% FEC	1 mA cm^{-2}	2 mA h cm^{-2}	CE ~75% Within 30 cycles	ACS Mater. Lett. 2019, 1, 254-259.
1 M $LiPF_6$ in EC/DEC/EMC (1/1/1 vol.)	1 mA cm^{-2}	1 mA h cm^{-2}	CE < 80% Dead at ~90 cycles	Chem. Eng. J. 2021, 414, 128928.
1 M $LiPF_6$ in EC/EMC (1/1 vol.) + 2 wt.% FEC	0.5 mA cm^{-2}	1 mA h cm^{-2}	CE ~90% Dead at 56 cycles	ACS Energy Lett. 2021, 6, 3170-3179.
1 M $LiPF_6$ in EC/EMC (3:7 by wt.) + 2 wt.% VC	0.5 mA cm^{-2}	1 mA h cm^{-2}	CE ~44% Within 40 cycles	Joule 2019, 3, 1662-1676.
1.2 M $LiPF_6$ in EC/EMC (3:7 vol.)	1 mA cm^{-2}	2 mA h cm^{-2}	CE ~60% Dead at ~15 cycles	Energy Storage Mater. 2019, 17, 284-292.
1.2 M $LiPF_6$ in FEC/EMC (3:7 vol.)	1 mA cm^{-2}	2 mA h cm^{-2}	CE ~94% Within 20 cycles	
1.2 M $LiPF_6$ in DFEC/EMC (3:7 vol.)	1 mA cm^{-2}	2 mA h cm^{-2}	CE ~96% Within 20 cycles	
1 M $LiPF_6$ in EC/EMC (1:1 vol.)	0.5 mA cm^{-2}	0.5 mA h cm^{-2}	CE ~84.4% Dead at 20 cycles	ACS Energy Lett. 2021, 6, 1839-1848.
1 M $LiPF_6$ in EC/EMC (1:1 vol.)+5% FEC	0.5 mA cm^{-2}	0.5 mA h cm^{-2}	CE ~94.1% Dead at 120 cycles	
1 M $LiPF_6$ in FEC/EC/DMC (2/9/9 vol.)	1 mA cm^{-2}	1 mA h cm^{-2}	CE ~80% Within 150 cycles	ACS Energy Lett. 2022, 7, 569-576.
1.2 M LiDFOB in EC/EMC (3:7)	/	/	CE ~95% Within 50 cycles	Energy Environ. Sci. 2018, 11, 2600-2608.
1 M $LiPF_6$ in EC/DMC (1:1) + 12 Mm $In(TFSI)_3$	1 mA cm^{-2}	1 mA h cm^{-2}	CE ~95% Within 160 cycles	Angew. Chem. Int. Ed. 2017, 56, 13070-13077.
1 M $LiPF_6$ in EC/DEC (1:1) + 0.15 M LiDFP	0.5 mA cm^{-2}	1 mA h cm^{-2}	CE ~95.2% Within 80 cycles	ACS Appl. Mater. Interfaces 2018, 10, 22201-22209.
1 M $LiPF_6$ in EC/DMC (1:2) + 2 wt% TTFEB	0.1 mA cm^{-2}	0.5 mA h cm^{-2}	CE ~96% Within 100 cycles	Energy Storage Mater. 2018, 11, 197-204.
1 M $LiPF_6$ in EC/DMC + 1 wt% dimethyl sulfate	0.5 mA cm^{-2}	1 mA h cm^{-2}	CE ~92% Within 160 cycles	ACS Appl. Mater. Interfaces 2017, 10, 593-601.
1 M $LiPF_6$ in FEC/DMC (1:1 v/v)	0.5 mA cm^{-2}	1 mA h cm^{-2}	CE ~93.25% Within 160 cycles	Energy Storage Mater. 2018, 15, 299-307.

Table S3. Cycling performance of Li metal batteries with ester-based electrolytes.

**Li(μm)‖Cathode**	**Electrolyte**	**N/P**	**Cycling performance**	**Ref.**
Li‖LFP	a-tFB	3	250 cycles, 90%	Our work

Li(5 mAh)‖ NCM622 (0.3 mAh cm^{-2})	1 M LiTFSI in FEC/1,2-dfBen	~16.67	300 cycles, 88.7%	ACS Appl. Mater. Interfaces, 2024, 16(46): 63628-63637.
Li(5 mAh)‖ LFP (0.2 mAh cm^{-2})		~25	1500 cycles, 80.0%	
Li‖NCM622 (5 mg cm^{-2})	0.8 M LiTFSI + 0.2 M LiDFOB – FEC/DEC/1,3-dFB (2:2:4 by vol)	/	300 cycles, 80%	Energy Storage Sci. Technol., 2024, 13(7): 2124.
Li(50)‖NCM811 (3.8 mAh cm^{-2})	1.25 M $LiPF_6$ in asymF-DEC/FEC/VC (8:2:0.5 v/v)	2.7	240 cycles, 80%	Angew. Chem. Int. Ed., 2020, 59, 14869-14876
Li(500)‖NCM811 (2.2 mAh cm^{-2})	$LiPF_6$, FEC, ETFEC, and PFPN with a molar ratio of 1:3.8:3.8:0.4	46.84	200 cycles, 85%	Adv. Energy Mater., 2022, 12, 2103360
Li(400)‖NCM811 (0.5 mAh cm^{-2})	1.0 M LiFSI in FEC: FEMC: DME (3:3:2 v/v) and 0.5 wt% LiNO	~165	500 cycles, 80%	Nano Energy 2024, 123, 109362.
Li(400)‖LCO (1.4 mAh cm^{-2})	1.2 M $LiPF_6$ in FEC: FDG (3:7 v/v)	~58.89	200 cycles, 84.2%	Nat. Commun. 2022, 13, 2575
Li(20)‖NCM811 (1 mAh cm^{-2})	2 M LiFSI in DTDL	~4.12	200 cycles, 84.0%	Chin. Chem. Lett. 2024, 109773.
Li(40)‖NCM523 (2.5 mAh cm^{-2})	1.3 M LiFSI/0.7 M LiTFSI/0.02 M $LiPF_6$ in DMC/1,2-dfBen	~3.3	100 cycles, 90.0%	J. Power Sources 2023, 559, 232631.
Li(50)‖NCM712 (3.6 mAh cm^{-2})	LiFSI:DMC:TFB (1:1.2:0.5 by molar)	~2.86	80 cycles, 80.0%	Adv. Funct. Mater., 2023, 33, 2212000
Li(50)‖LCO (3.5 mAh cm^{-2})	2 M LiPF6 in DMC–FEC–FEMC (3:1:1)	~3	200 cycles, 76.9%	Adv. Energy Mater., 2023, 13, 2301396
Li‖LFP	0.1 M LiTFSI in FEC-FB (2 :8, by volume)	4	160 cycles, 80%	Angew. Chem. Int. Ed. 2024, 63, e202319090
Li‖NCM811		3	150 cycles, 80%	
